# Ultrasound-based artificial intelligence for breast lesion classification

**DOI:** 10.3389/fonc.2026.1759194

**Published:** 2026-02-26

**Authors:** Ting Ma, Zhen Wang, Jian Dong, Yuhang Cheng, Huan Zhao, Xinwu Cui

**Affiliations:** 1 Department of Medical Ultrasound, Tongji Hospital, Tongji Medical College, Huazhong University of Science and Technology, Wuhan, China; 2 Department of Medical Ultrasound, The First Affiliated Hospital of Shihezi University, Shihezi, Xinjiang, China

**Keywords:** artificial intelligence, breast lesion, convolutional neural networks, deep learning, ultrasound

## Abstract

Breast cancer is the most prevalent cancer among women. Early and accurate screening is crucial for improving patient outcomes. Ultrasound is a valuable diagnostic tool, particularly for dense breasts, yet its efficacy can be limited by operator dependency and interpretive variability. Artificial intelligence (AI) has shown significant potential to enhance the accuracy and efficiency of breast ultrasound. However, translating AI from research to clinical practice remains challenging due to several persistent gaps: the lack of robust clinical validation for generative AI in image enhancement; insufficient focus on AI for diagnosing non-mass lesions, which constitute a notable proportion of malignancies; and limited multi-center effectiveness data for commercial computer-aided diagnosis systems. This narrative review synthesizes recent advancements in AI for breast ultrasound and provides a critical, multifaceted analysis that integrates technological evolution, clinical-translation challenges, and implementation frameworks. Importantly, it highlights pervasive methodological limitations, such as small sample sizes, retrospective single-center designs, and inadequate external validation, that often lead to overestimation of real-world AI performance. By offering both actionable insights and a cautionary perspective, this review aims to guide the rigorous, evidence-based translation of AI into clinically viable tools.

## 1 Introduction

Breast cancer (BC) is a heterogeneous disease caused by the complex interaction of multiple factors such as genetics, lifestyle, and hormones (1). Global cancer-related reports in 2022 proposed 2,308,897 new cases of BC in females, which is behind lung cancer as the second most prevalent cancer worldwide (11.6%) (2). Most women with early-stage BC may have the opportunity to opt for breast-conserving surgery, a less invasive option that offers a better prognosis (3). Advanced BC may metastasize to distant tissues and organs such as the lungs, spine, and liver, thereby limiting patients’ access to optimal treatment and ultimately leading to a grim prognosis. The survival rate of BC patients with advanced cancer or distant metastases is only three years, and a five-year survival rate of approximately 26% alongside a terrible quality of life (4, 5). Despite a recent decline in mortality rates, BC remains a significant cause of death among women globally, with an estimated 665,684 deaths annually. It accounts for 6.9% of all cancer-related deaths in women, which seriously threatens women’s health and global public health security (2, 6). Therefore, early and accurate screening to assess breast lesions, understand the trend of BC changes, and guide the early treatment of BC patients is the key to improving patient outcomes and prognosis.

The diagnosis of breast lesions involves a comprehensive evaluation of clinical symptoms, physical findings, tumor marker tests, radiological features, and pathology tests. Currently, mammography and ultrasound are widely used as the primary imaging tools for breast lesion screening. Ultrasound and screening mammograms exhibit similar sensitivity, specificity, cancer detection rates, and biopsy rates. However, ultrasound screening has a higher rate of detecting invasive cancers (7, 8). The high contrast resolution of ultrasound on soft tissue makes it independent of gland type and avoids the requirement for breast compression, making it more suitable for early screening in relatively young women with dense glandular types (9, 10). Recently, advancements in automated breast ultrasound systems have addressed the operator-dependent limitations of conventional handheld ultrasound devices by providing 3D formatted images, which improve the diagnostic accuracy (11). In addition, ultrasound has demonstrated superior diagnostic capabilities compared to other imaging methods in detecting axillary lymph node metastases in patients with BC. Its benefits such as the absence of ionizing radiation, the ability to guide cytological and histological diagnostic studies, and the support of surgical interventions, have contributed to the growing use of ultrasound in the screening and descriptive breast lesion classification.

The identification of benign and malignant classification of breast lesions is a crucial step following lesion screening and an essential component of the clinical diagnosis and treatment of BC. Currently, the potential role of artificial intelligence (AI) algorithms in ultrasound has been widely explored (12). AI, a branch of data science, can extract numerous image features that are invisible to the naked eye based on the radiomics of ultrasound (13). The AI system provides a predictive modeling train on high-throughput quantitative features extracted from radiological data through image processing, segmentation, and feature extraction. Automated breast ultrasound systems allow for the standardized acquisition of comprehensive breast images, mitigating operator-dependent variability and reducing interference from extramammary factors that can compromise image quality (14). A complete standardized AI-assisted breast ultrasound procedure takes about two minutes, reducing interpretation and reporting time by approximately 40%, maximizing the efficiency of the sonographer’s task (15, 16). AI feeds through the amount of image data from various modalities to provide accurate malignant risk assessments of the breast lesion, which improves the diagnostic accuracy of less-experienced sonographers and frees up physician resources. Therefore, in-depth research on AI in breast ultrasound is essential. Despite the valuable contributions of prior reviews, several persistent gaps impede the translation of AI from research to clinical practice. Specifically, three underexplored yet critical areas include: (I) the clinical validation gap for generative AI (GenAI), particularly in ultrasound image enhancement and microbubble localization; (II) the diagnostic challenge of non-mass lesions, which represent 15–20% of breast malignancies but are often overlooked in AI studies; and (III) the lack of multi-center performance data for commercial computer-aided detection and diagnosis (CAD) systems, limiting their generalizability and clinical trust. This review is positioned to address these gaps by not only synthesizing technical advancements but also systematically examining translational pathways and implementation barriers.

While recent reviews have advanced breast cancer diagnostics, critical knowledge and translational gaps persist specifically in the application of AI to breast ultrasound. Systematic analyses have meticulously evaluated the promise of emerging imaging modalities like hyperspectral imaging for CAD, and broader assessments have critiqued the translational pathway of multiple novel techniques from innovation to clinical application (17, 18). However, these studies either focus on alternative imaging technologies or provide a high-level overview across modalities, leaving a focused, in-depth synthesis of AI’s evolution and integration within the established, first-line screening tool, breast ultrasound, less comprehensively addressed. Furthermore, discussions on socioeconomic barriers to prevention highlight crucial contextual challenges in healthcare delivery, underscoring the need for AI solutions that are not only technologically robust but also adaptable to diverse clinical and resource settings (19).

Consequently, this narrative review aims to fill these voids by concentrating on the intersection of artificial intelligence and breast ultrasound imaging. We posit that despite ultrasound’s primary role in screening, particularly for dense breasts, a focused analysis of how AI models are evolving to interpret its imagery, overcome its operator-dependency, and integrate into clinical workflows is urgently needed. Key underexplored areas include the clinical validation of generative AI for image enhancement, the diagnostic handling of challenging non-mass lesions often missed in AI training sets, and the real-world, multi-center performance data of commercial AI-CAD systems—gaps that are pivotal for translation but not centrally addressed in prior syntheses.

To bridge these identified gaps, this review not only synthesizes state-of-the-art AI applications but also provides a critical and multifaceted analysis. This analysis scrutinizes AI model evolution, examines the methodological strengths and weaknesses of clinical translation studies, and dissects the implementation barriers of commercial frameworks in light of often-overlooked factors like dataset bias and generalizability deficits. We outline the current state of AI in breast ultrasound, with a particular focus on its application in lesion classification and malignancy grading. Beyond summarizing existing research, we critically examine translational challenges, explainable AI frameworks, and the integration of commercial CAD systems into clinical workflows. By synthesizing technical advancements with a critical appraisal of real-world diagnostic challenges and the methodological constraints of existing studies, this review provides a balanced perspective that bridges the gap between AI research and the requirements for robust, generalizable clinical practice. It offers actionable insights not only on technological trends but also on the translational pathways and implementation hurdles. Critically, it distinguishes between the promising efficacy demonstrated in research settings, often reliant on single-center, retrospective data, and the robust evidence required for clinical viability. This distinction provides a more realistic roadmap for researchers and clinicians, highlighting that the development and deployment of reliable AI solutions in breast ultrasound necessitate a focus on prospective validation and generalizability, alongside their practical utility.

## 2 Methodology and scope of this review

This work is structured as a critical narrative review. Its primary objective is to identify, synthesize, and critically analyze the current state of artificial intelligence in breast ultrasound, with a focused aim of bridging the translational gaps between research prototypes, commercial systems, and clinical practice. Unlike a systematic review with a narrow PICO question, this review adopts a broader perspective to provide a comprehensive, interdisciplinary analysis of technological evolution, clinical applications, and implementation challenges.

To construct this narrative, we undertook a structured but non-exhaustive literature search to identify foundational and impactful works. Electronic databases, including PubMed, IEEE Xplore, and Google Scholar, were queried using combinations of keywords such as “artificial intelligence”, “deep learning”, “convolutional neural network”, “breast ultrasound”, “breast lesion classification”, “computer-aided diagnosis”, “radiomics”, and “generative AI”. Given the review’s translational focus, we prioritized peer-reviewed original research, high-impact review articles, clinical validation studies, and key reports on commercial CAD systems. Special emphasis was placed on identifying literature that directly informed the three critical gaps outlined in the introduction: (1) clinical validation of generative AI, (2) diagnosis of non-mass lesions, and (3) multi-center performance of commercial systems. No formal quality scoring tool was applied, but studies were critically appraised based on sample size, study design, validation method, and clarity of reported metrics.

The identified literature was analyzed thematically rather than quantitatively. The synthesis is organized to first establish the technological foundations, then examine applications and clinical decision-making, evaluate commercial systems and translational readiness, and finally propose a future roadmap. This structure is designed to facilitate a critical examination of the field’s progress and persistent bottlenecks from multiple angles.

## 3 Overview of artificial intelligence technologies

### 3.1 Artificial intelligence

The concept of AI was first introduced to the public by John McCarthy in 1956 during the Dartmouth Conference. AI aims to develop systems that assist with tasks requiring human expertise. In medical imaging, AI excels at analyzing complex image data to identify patterns and support diagnostic decisions, offering tools like radiomics for quantitative feature analysis (20).

AI infrastructure layer supported by machine learning (ML) core algorithms and AI infrastructure frameworks supports and realizes AI applications by learning the internal laws of data and training models (21). For medical image analysis, ML and its advanced subset, deep learning (DL), are the most relevant AI paradigms. Convolutional neural networks (CNNs), a specialized DL architecture, are particularly pivotal for ultrasound image interpretation. Their key characteristics are summarized in **Table 1**.

**Table 1:** The key differences between machine learning, deep learning, and convolutional neural network.

AI technology	Brief definition	Core technology	Data requirements	Representative model	Application of breast ultrasound	Advantages and limitations
ML	An algorithm framework for learning patterns from data	Supervised Learning Unsupervised Learning Feature Engineering	Medium-scale annotation data	RF SVM	Classification of lesion echo features	Advantages: Strong interpretability Limitations: Relying on manual feature extraction makes it difficult to handle complex patterns
DL	AI branch driven by multi-layer neural networks	Back-propagation Automatic feature extraction	Large-scale annotated data	ResNet DenseNet	Dynamic analysis of ultrasound video	Advantages: end-to-end learning Limitations: High computational resource requirements and poor model interpretability
CNN	DL architecture is designed specifically for images, preserving spatial relationships	Convolutional kernels Pooling operations	Large-scale image data	AlexNet U-Net	Microcalcification detection, Classification of non-mass lesions	Advantages: High efficiency in image feature extraction Limitations: Sensitive to ultrasound speckle noise, requiring standardized image input

ML, Machine Learning; DL, Deep Learning; CNN, Convolutional Neural Network; RF, random forest; SVM, Support Vector Machine; ResNet, Residual Network.

AI opens unprecedented possibilities for medical research, particularly in complex data analysis, pattern discovery, and predictive modeling. AI big data analytics are forming an emerging field through effective integration with medical imaging, especially noninvasive advanced imaging analysis, known as radiomics. AI analyzes lesion features extracted from large volumes of digital quantitative information from radiological imaging to provide predictive models. AI algorithms are trained by extracting and analyzing markers, such as pathology test results and genetic microcoding, from patient diagnosis or prognosis. The AI system processes image data from various imaging modalities to obtain the output information and continually undergoes self-correction and re-learning to make diagnostic and treatment decisions (22). Imaging physicians, after scrutinizing numerous image data for diagnosis, may suffer from fatigue, potentially resulting in missed diagnoses or misdiagnoses. AI has shown significant potential in revolutionizing medical diagnostics, offering high accuracy and efficiency in identifying diseases such as cancer at early stages and providing personalized treatment plans by analyzing vast amounts of medical data. to mimic the work of humans in medical image processing for a long period and make the correct diagnosis, improving the efficiency of radiologists by reducing interpretation time (23). AI-based radiomics is increasingly utilized in diagnostic ultrasound. The pre-training of AI models on extensive image datasets is a critical step in leveraging AI for radiomics, particularly in diagnostic ultrasound, as demonstrated by recent advancements in breast ultrasound and liver fibrosis staging. The pre-training of models extracts deep features from rich ultrasound images, which in turn can help identify new biomarkers or develop new criteria for diagnosing a specific disease. These trained models can assist clinicians in interpreting ultrasound images and automatically provide diagnostic results or recommendations, which not only saves time and improves access to healthcare but also optimizes ultrasound diagnosis.

In 2022, generative AI (GenAI) emerged as a transformative branch of AI, capable of creating new data instances that resemble the training distribution (24). Within breast ultrasound, GenAI holds immediate promise for addressing two pivotal challenges: image quality enhancement and data scarcity (25, 26). For image enhancement, GenAI models, particularly those based on Generative Adversarial Networks (GANs) or diffusion models, can be employed to reduce characteristic speckle noise, improve image resolution, and synthesize one imaging modality from another. These applications aim to improve lesion conspicuity and feature extraction, directly impacting diagnostic confidence. For data augmentation, GenAI can generate high-quality, annotated synthetic breast ultrasound images. This is crucial for expanding training datasets, balancing class distributions, and simulating rare or challenging presentations like non-mass lesions, thereby mitigating the data-hungry nature of deep learning models.

Currently, GenAI is increasingly being used in healthcare to inform clinical service functions. GenAI can improve diagnostic accuracy through knowledge acquisition for screening and diagnosing diseases. While GenAI is still in the process of being fully realized, the healthcare industry has already seen significant strides in its application, with 62% of healthcare and life science executives implementing GenAI solutions and 74% experiencing investment returns in at least one use case. Continued research and development are essential to deepen the integration of GenAI in healthcare, particularly in areas such as automated services, to further revolutionize drug development and personalized patient care.

However, a pronounced chasm separates this technical potential from validated clinical utility. The majority of GenAI applications in breast ultrasound are proof-of-concept demonstrations reliant on small, often idealized datasets, remaining far from clinical readiness. The most critical evidence gap, as highlighted earlier, is the near absence of robust, multi-center clinical trials. Such trials are essential to determine if GenAI-driven enhancements yield measurable, reproducible improvements in diagnostic accuracy and workflow efficiency across diverse clinical environments, or if reported gains are specific to constrained experimental settings.

The evolutionary path forward for AI in breast ultrasound points toward systems with enhanced multimodal integration and clinical context-aware decision support, moving beyond single-modality, task-specific classifiers. Rather than pursuing the broad and distant goal of Artificial General Intelligence (AGI), the near-term focus is on developing specialized AI systems that can emulate a radiologist’s integrative reasoning process. Such systems aim to automatically fuse and interpret diverse data streams available in a clinical setting. This includes correlating features across multiple ultrasound modalities from the same exam and incorporating relevant clinical context from electronic health records.

The technical foundation for this next stage lies in advancing multimodal learning architectures, cross-modal representation learning, and explainable AI (XAI) frameworks that make the integrated decision-making process transparent. By doing so, AI has the potential to evolve from an image analysis tool into a comprehensive clinical decision-support partner. It could provide a unified risk assessment, suggest differential diagnoses, and flag inconsistencies, thereby assisting in complex case evaluation and management planning.

This progression from an image analysis tool to a clinical decision-support system underscores the translational goal of AI: to augment and standardize the radiologist’s cognitive process, thereby enhancing the consistency and accuracy of diagnostic outcomes across diverse clinical settings and levels of expertise. Achieving this requires concerted efforts in creating large, curated, multimodal datasets and developing robust validation frameworks to ensure these advanced systems are safe, effective, and trustworthy in real-world clinical workflows.

### 3.2 Machine learning

ML is an emerging field that combines computer science and statistics. Unlike classical AI programming, which executes algorithms to produce results, ML enables computers to learn from input data without being explicitly programmed to use the dataset and associated outputs to generate algorithms that can describe the relationship between the two (27, 28).

#### 3.2.1 Traditional machine learning tasks

Supervised learning, as the most dominant ML approach, focuses on inferring algorithms from data features and target outputs, back-propagating from target outputs to predict the accuracy of the algorithms. The algorithm optimization process involves assessing model accuracy on the new dataset, which subsequently guides necessary adjustments to the algorithm. Supervised Learning requires a large amount of well-labeled data for classification to train the algorithm (29).

Unsupervised learning consists of unlabeled data presented to an algorithm to find implicit patterns. Unsupervised learning techniques, such as clustering, correlation, and anomaly detection, focus on detecting centralized patterns of correlation between data and categorizing the individual data in the dataset (30). Common algorithms in this field include clustering review metrics, principal component analysis, and independent component analysis.

Semi-supervised learning, particularly in medical image segmentation, is widely adopted as it leverages both labeled and unlabeled datasets to enhance the performance of models, often outperforming purely unsupervised approaches. Due to the time-consuming and laborious nature of labeling large datasets, a practical approach is to label a small subset for training, enabling the model to classify the remaining unlabeled images. This labeled dataset subsequently serves to train a new, functional model, and the labeled classification of large data and iterative refinement of the model is performed over time (31, 32).

#### 3.2.2 Common algorithm models in machine learning

ML algorithms represented by linear regression, logistic regression (LR), decision tree (DT), random forest (RF), and support vector machine have been proficiently applied in medicine.

**Figure 1:**
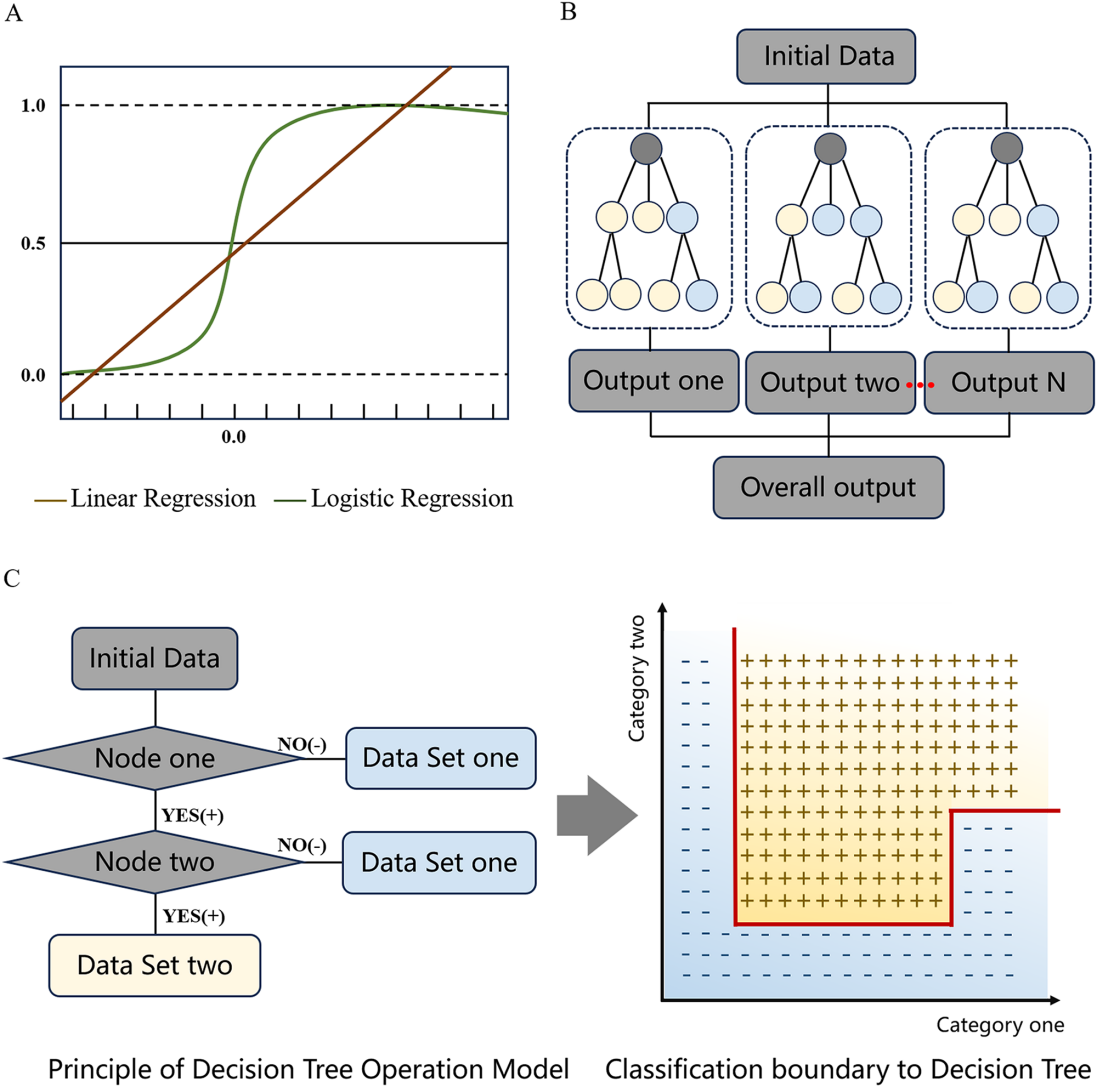
Schematic diagram of common algorithm models in traditional machine learning. **(A)** Comparison of linear regression and logistic regression, illustrating their decision boundaries; **(B)** Schematic of the Random Forest structure, demonstrating parallel tree processing; **(C)** Decision tree flowchart with classification branches and resulting data separation in feature space.

Linear regression is based on the assumption of a linear relationship and minimizes the squared difference between predicted and actual values by finding the most suitable straight line for the data (see **Figure 1A**). Linear functions are simple, intuitive, and fast, but they poorly fit nonlinear data and are sensitive to outliers.

LR is a statistical model for binary classification problems, mainly used to find the probabilistic relationship between data features of the input variables and the target outcome. (see **Figure 1A**). The LR model can be flexibly implemented to analyze the effect of binomial or polynomial features on the target output. It is simple and computationally fast with good interpretability, but only applicable to linearly separable data and needs to be extended for multiclass problems.

DT accomplishes the dual task of classification and regression. It learns mapping relationships from data features to outputs by DT using a binary-like recursive partitioning method. DT splits the dataset layer by layer based on specific features to form a series of rules that combine to resemble a tree (see **Figure 1C**).

RF belongs to the category of integrated learning, which performs model operations by generating multiple DT (see **Figure 1B**). Each DT is created by subsampling features, and the majority of the results calculated within it are used as the final class prediction of the model. This approach enhances the accuracy and stability of the predictions. But it does not have the ability to handle difficult samples.

#### 3.2.3 Achievements and development of traditional machine learning

ML analyzes and processes information by extracting lesion features and provides the final algorithm to assist radiologists to detect and analyze lesions. Traditional supervised learning techniques such as LR, RF in ultrasound image analysis have made significant progress. Their applications mainly focus on the following aspects: (I)image feature extraction: automatically extracting key features in ultrasound images, such as tumor composition, echo, edge, shape, and texture, etc., by ML algorithms; (II)image classification and diagnosis: using extracted lesion features to assist doctors in disease diagnosis; (III)radiomics: combining ML techniques to extract the amount of high-throughput features for disease prediction and classification.

Conventional ML was developed for its accuracy and reliability in ultrasound image analysis. ML, especially RF algorithmic models, can automatically extract features from images, reducing the reliance on expert experience and making ultrasound image analysis more objective and standardized. A study of 895 ultrasound images found that the ultrasound-based RF algorithm performed best compared to the remaining algorithms in classification (ACC: 0.90, AUCPR: 0.90, F1 score: 0.83) (33). The ML model demonstrated excellent stability and accuracy with its powerful feature selection capability and classification performance.

#### 3.2.4 Limitations of traditional machine learning

Nonetheless, research into ML algorithms at this juncture encounters obstacles like limited dataset size and intricate algorithm implementation. The continuous development of ultrasound imaging technology has opened up a new direction for improving imaging resolution, but further research and optimization are needed to overcome the existing challenges.

### 3.3 Deep learning

DL cognitions features and patterns from data by constructing neural network models with multiple layers. It can autonomously “learn” from erroneous algorithmic outputs without human intervention, enhancing algorithmic stability and accuracy (34, 35). Compared to traditional ML methods, DL offers greater feature extraction capabilities and higher model complexity. That is capable of handling large-scale data and complex tasks.

#### 3.3.1 Common neural network algorithm models for deep learning

DL-related algorithmic models mainly include Deep neural network (DNN), Recurrent neural network (RNN), Generative adversarial networks (GAN), and Convolutional neural network (CNN).

DNN is one of the most basic models of DL and usually includes an input layer, a hidden layer, and an output layer. The hidden layer can have multiple layers, each containing multiple neurons (36).

The CNN calculates error through a loss function as the algorithm runs, applying a backpropagation algorithm to instruct the machine on how to change its internal parameters and continually updating the connection weights to adjust them to better fit the relationship between the input data and the interested outputs (37). The algorithms provide positive feedback to reinforce the desired output. The relationship between input features and outputs is learned through layers of incremental computation and optimization, gradually improving the ability to process the input data and the accuracy of the outputs (38, 39).

RNN takes the input of the current moment and the output of the previous moment simultaneously as the overall input of the current moment, utilizing a recurrent structure and introducing a temporal dimension. RNN automatically learns temporal dependencies in sequential data, efficiently processing sequential inputs of data like language, voice, text, and time series (37, 40, 41). Additionally, cyclic highway networks have further developed long- and short-term memory architectures and have solved the gradient problem of traditional RNN to better capture long-term dependencies in sequences.

As the name suggests, GAN is a DL model consisting of two neural networks, the generator and the discriminator, competing and cooperating, which belongs to the category of unsupervised learning. In radiology, GAN can synthesize realistic medical images (42).

#### 3.3.2 Achievements and development in deep learning

DL has been developed significantly in medical imaging, where DL techniques can directly encode mappings. A meta-analysis evaluating the diagnostic accuracy of DL algorithms found that they have high diagnostic accuracy for a wide range of common diseases in terms of X-ray, ultrasound, CT, and MRI. Its application in ultrasound is also gradually transforming traditional ultrasound diagnostic methods (26, 43, 44).

Ultrasound images can be corrupted by scattering noise, hindering disease diagnosis and treatment progression. DL techniques hold the potential to enhance the clarity of ultrasound images and diminish noise levels. Luijten B. et al. used adaptive signal processing algorithms to constrain DNN, which can efficiently learn and perform fast, high-quality ultrasound beam formation using less training data (45). Liu et al. developed a CycleGAN model based on bi-directional universal mapping that enables style migration between the noisy data domain with scattering and the noisy data domain without noise, and demonstrated the superiority of the model in evaluating quantitative scattering signals as well as noise reduction and detail preservation, thus improving the quality of the ultrasound images (46). Sudharson S. et al. developed an integrated classification model with high accuracy (ACC: 0.95, 95% CI: 0.94 - 0.96) on ultrasound images with noisy speckles (47, 48).

In breast ultrasound imaging, DL technology has proven to be a powerful, efficient, and highly accurate tool that can save examination time, improve lesion detection, and potentially compensate for physician experience deficits. A single-center retrospective study with 637 breast ultrasound images found that the DL model trained on small samples had a diagnostic AUC of 0.84 for breast lesions (SEN: 0.84, SPE: 0.80, PPV: 0.32, NPV: 0.97). The performance was comparable to the diagnostic results of radiologists and better than that of trained medical students. AI-assisted classification and diagnosis of breast disease may improve diagnostic accuracy for novice physicians (49).

DL has shown remarkable results in color Doppler imaging, shear-wave elastography (SWE), and ultrasonography. In breast lesion classification, color Doppler neural network models can achieve highly consistent interpretation results with experienced radiologists (AUC: 0.98 vs 0.95) and potentially automate routine characterization (50). A GAN-based microbubble localization model can detect microbubbles with an accuracy of 0.98, enabling high-precision localization of microbubbles in ultrasound contrast agents. The discovery of this model is crucial for the development of generalized solutions for different imaging conditions and types of biological tissues (51). Fei et al. investigated the synthesis of elasticity images via a multi-scale elasticity image synthesis network, and diagnostic tests demonstrated that the classification performance of the synthesized elasticity images was similar to that of the diagnostic performance of real elasticity analysis of semi-quantitative data (52).

Beyond basic screening and classification, DL models can determine whether the postoperative lesion staging of ductal carcinoma *in situ* diagnosed by preoperative hollow-core needle biopsy will be upgraded or not. Qian et al. retrospectively analyzed 360 images of confirmed ductal carcinoma *in situ* using a DL model applied to ultrasound images. The AUC of four DL models ranged from 0.72 to 0.80, with ResNet and Inception showing a more accurate diagnostic value (53). The ultrasound-based DNN prediction model can effectively predict patients with ductal carcinoma *in situ* who may be classified as upgraded after surgery, guiding the clinic to intervene as early as possible and make more accurate decisions.

#### 3.3.3 Limitations of deep learning

With the ongoing technology development, the research and application of DL in medical imaging are moving in a deeper and wider direction. A fundamental constraint is the reliance on limited, high-quality labeled datasets, which not only restricts model training but, more critically, severely compromises generalizability. This issue of generalizability is fundamentally a problem of domain shift. AI models trained on data from one institution often experience significant performance degradation when applied to data from another, due to differences in ultrasound machine vendors, imaging protocols, operator techniques, and patient population characteristics. This equipment and acquisition heterogeneity represents a major, often under-appreciated, barrier to clinical deployment. Merely expanding the dataset size through multi-center collaboration is insufficient unless it explicitly addresses and quantifies this variability. To address this, expanding datasets through multi-center collaboration is essential, and this effort should be guided by a push towards standardized imaging protocols and annotation criteria to maximize data utility and minimize confounding technical heterogeneity.

### 3.4 Convolutional neural networks

**Figure 2:**
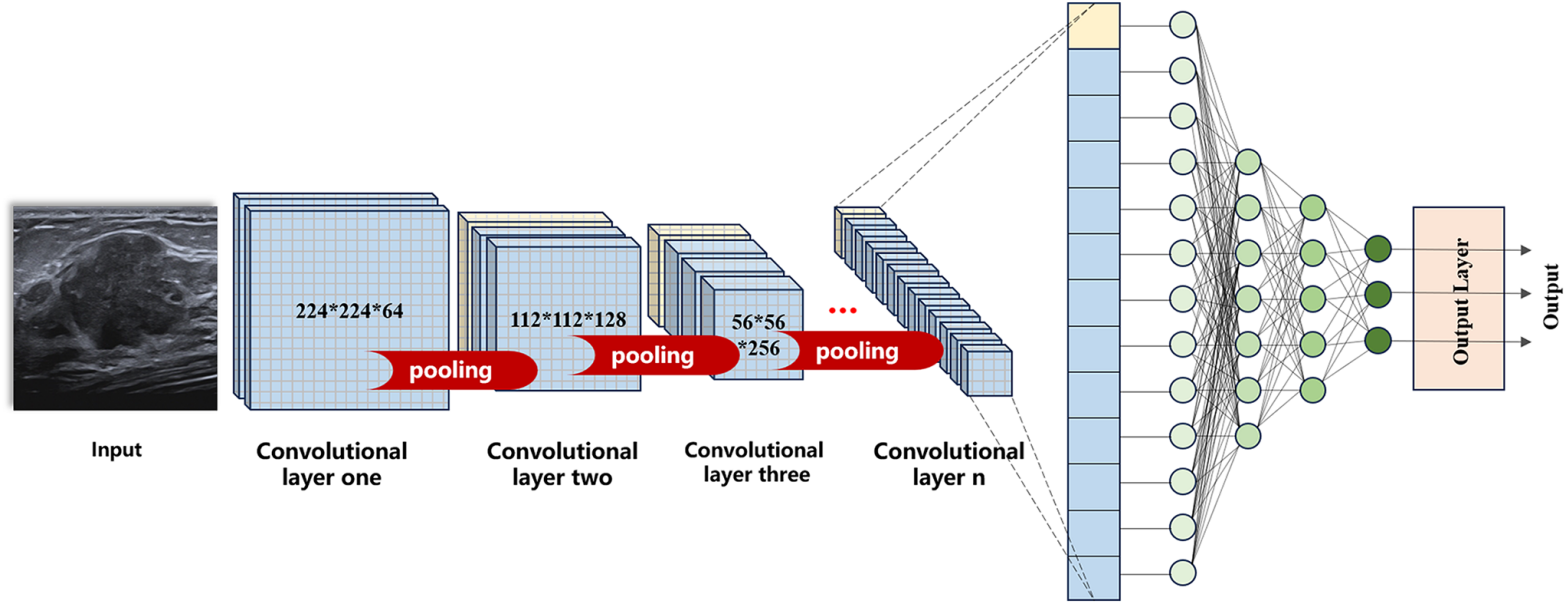
Convolutional neural network operation mode diagram.

In image recognition tasks, each input to an artificial neural network corresponds to a pixel, without considering the connectivity between nodes in the layer, potentially losing the spatial context of image features in practical applications (54).CNN, a key subset of DL, preserves the spatial relationships between image pixels through the combinations of input, convolutional, pooling, fully connected, and output layers to automatically learn features and patterns in images(see **Figure 2**) (32).

#### 3.4.1 Convolutional neural network operations

Convolutional operations are popular in edge detection, sharpening, and blurring tasks (55). CNN feeds the information from the image to some specific nodes in the next layer of nodes through convolutional filters, extracting various features of the input image at different levels, thus preserving the extraction of information from the feature space and generating the feature maps. The pooling layer reduces overfitting and decreases the in-plane dimensionality of the feature mapping. By sampling the output of the convolutional layer at each stage to decrease the resolution of the feature map, the model’s parameters are reduced while preserving the fundamental characteristics of the source image (28). As the new matrix feature map is continuously presented and the pooling layer is run, the extracted features become more abstract.

The fully connected layer maps the features extracted by the convolutional and pooling layers to the derived final output of the model. The final feature image is compressed from its matrix representation and fed into a feed-forward neural network, which classifies the image based on the extracted feature information such as lesion texture and edges.

#### 3.4.2 Strengths of convolutional neural networks

In a regular neural network, all neurons in each layer are randomly connected to all neurons in the subsequent layer. In practical CNN applications, obtaining high-quality, large-scale labeled data for training is often challenging due to difficulties such as ensuring data diversity, maintaining data quality, and managing high annotation costs. How to improve the model performance with limited data and parameters is an urgent challenge. However, neurons in each layer of a CNN do not form a one-to-one connection relationship with all neurons in the next layer, but rather feed the information of the image to a small portion of specific neuron nodes in the nodes of the next layer through a convolutional filter kernel (56). Only this small subset of specific neuron nodes needs to be trained. This drastically reduces the number of parameters that need to be learned, making the models more efficient to train and less prone to overfitting, especially with limited medical image data (29).

CNN enables fine-tuning algorithmic models that have been trained in other projects for new tasks and new datasets to rapidly reach model maturity, commonly known as “transfer learning”. Numerous studies have developed new models based on pre-trained CNN models, such as ImageNet, AlexNet, GoogleNet, VGGNet, DenseNet, and ResNet (57, 58). Transfer learning involves leveraging a pre-trained model as a feature extractor, continually extracting features such as edges and curves for object detection and image classification and then quickly training with the new task dataset. The fully connected layers are substituted by the new set, generating the target output for the new task. Transfer learning can reduce training parameters and decrease the data requirements for developing CNN models by reusing the pre-trained model parameters (32).

#### 3.4.3 Achievements and development of convolutional neural networks

In the medical field, CNN reduces the number of parameters by optimizing connections, improving computational efficiency. It can rapidly adapt to new diagnostic tasks based on existing pre-trained models through transfer learning techniques. Improving the interpretability of CNN is a research priority to enhance the trust of medical professionals in the models and to promote their application in key areas such as clinical diagnosis.

Numerous new CNN-based models demonstrate enhanced capabilities in feature extraction, classification, identification, and the detection of cancer in breast ultrasound images. Cao et al. conducted an analysis comparing the effectiveness of AlexNet, ZFNet, VGG16, GoogleNet, ResNet, and DenseNet models in the screening and classification of breast lesions. They found that the models have good diagnostic performance and DenseNet attained the highest accuracy of 0.85 for lesion classification and a predefined region of interest (ROI) of 0.88 (59). The classical LeNet architecture, which addresses the issue of missing linear units and enhances the discriminative ability of extracted features, achieved an accuracy of 0.90 for recognizing benign and malignant classification of breast ultrasound images (60). Alaa et al. also found that the optimized CNN model exhibited higher accuracy (61). The development of CNN video models has overcome the limitations of static ultrasound images. 3D ResNet-50 and KamNet can identify more detailed spatial and temporal information to accurately classify breast lesions, thereby improving the clinical diagnosis of BC (62, 63). Jarosik P. et al. investigated the potential for creating a DL breast lesion classification model using raw RF ultrasound data and proposed a CNN model-based breast lesion classification method. is capable of automatically handling RF ultrasound signals and is trained on both the RF signals and their envelope samples. The classification performance substantially surpasses that of various other parametric classifiers (AUC: 0.77 vs. 0.64). This study expands the application of AI in breast ultrasound by uniting the model with new ultrasound techniques.

#### 3.4.4 Limitations of convolutional neural networks

Nevertheless, CNN faces challenges in medical applications. First, the computational complexity of CNN is high, requiring a lot of computational resources and time. How to optimize the structure and algorithm of CNN to improve computational efficiency is an important direction for future research. Furthermore, malicious attacks and noise interference can lead to incorrect outputs from CNNs. Enhancing the security and robustness of CNN to ensure its reliability in medical applications is an important research topic that needs to be solved.

## 4 Applications

### 4.1 Radiomics

Radiomics combines large data analysis technology with medical imaging, which is mainly divided into five steps: medical image acquisition, ROI segmentation, feature extraction, feature selection, model building, and validation. Radiomics can acquire X-ray, US, CT, MRI, and even biopsy slice images. The regions related to the lesion in the image are summarized and segmented for further feature extraction. Next, radiomics like signal intensity, lesion shape, size, and texture features are extracted from ROI regions. Features with good repeatability, stability, and independence are selected by feature selection methods, such as minimum absolute contraction. Finally, model building and testing of independent samples were performed by common statistical methods and advanced ML strategies (64).

The clinical imperative of radiomics extends beyond feature extraction. Its core promise lies in translating complex quantitative data into objective, actionable biomarkers that can resolve diagnostic ambiguity in equivocal lesions, provide prognostic insights complementary to histopathology, and ultimately enable more personalized and confident patient management decisions.

Radiomics features mainly describe the heterogeneity of the internal structure of the lesions, and statistical features quantify the grayscale distribution and texture patterns of the images. By extracting these features, feature vectors can be constructed for subsequent ML algorithms for classification and diagnosis.

A critical challenge in radiomics is feature reproducibility. Extracted features are highly sensitive to variations in image acquisition parameters, pre-processing steps, and segmentation methods. Without rigorous standardization across centers, the generalizability of radiomics models is fundamentally limited. Many published studies fail to adequately address this issue or to perform validation on fully independent, external datasets, which is essential to assess true clinical applicability.

Consequently, the external validation of radiomics models on completely independent datasets, preferably from institutions using different equipment and protocols, is not merely a best practice but a prerequisite for assessing true clinical applicability. Many published high-performance models fail this critical test, their accuracy metrics reflecting optimistic bias inherent to single-center, retrospective studies. Rigorous external validation serves as the primary means to expose and quantify the impact of domain shift.

The development of radiomics has enabled the close integration of engineering intelligence with imaging medicine, presenting unprecedented opportunities for medical diagnosis and research. In disease diagnosis, radiomics can dig out hidden subtle information, objectively assess inter- and intra-tumor heterogeneity through spatial distribution, and assist physicians in more accurately determining the type, stage, and prognosis of diseases (65, 66). In treatment planning, radiation therapists determine the irradiation range and dose more accurately based on the detailed information extracted from images, optimize the automatic planning process and dosimetric trade-offs, and improve the therapeutic efficacy while reducing damage to the surrounding healthy tissues (67, 68). In disease monitoring and efficacy assessment, real-time tracking of disease progression and evaluation of treatment efficacy through image analysis allows for early detection of signs of recurrence or assessment of the effectiveness of treatment, leading to adjustment of treatment strategies.

The applications of radiomics in BC mainly include both lesion classification and treatment outcome prediction. Lesion classification involved differentiating benign and malignant lesions, molecular subtypes, and other clinicopathologic indices, including the status of the anterior sentinel lymph nodes. As a whole, radiomics is currently a popular direction expected to improve the accuracy of the diagnostic classification of breasts, with ultrasound-associated radiomics developing particularly rapidly (69).

#### 4.1.1 B-mode ultrasound

Radiomics, when applied to B-mode ultrasound (B-US), enhances the accuracy of breast lesion classification by extracting a multitude of quantitative features from ultrasound images. This is supported by frameworks that utilize elastographic features, quantitative ultrasound parametric images, and computer-aided diagnosis systems to improve the efficiency and accuracy of lesion segmentation and classification.

In patients with dense breast tissue, the ultrasound can differentiate cystic solidity within the lesion and provide important information regarding the breast lesions classification. The Breast imaging reporting and data system (BI-RADS) provides a standardized digital presentation of malignancy risk in breast lesions. A clear delineation of the probability of malignancy from zero to greater than ninety-five percent is achieved through the six classifications of breast lesions. The BI-RADS and final assessment greatly reduce the individualized ambiguity in clinicians’ understanding of the recommendations of the imaging report (14, 70). For lesions in BI-RADS-3, further short-term follow-up is recommended, whereas for lesions in BI-RADS-4a or higher, immediate biopsy is recommended for diagnosis. The revised BI-RADS may offer the possibility of downgrading certain benign lesions from BI-RADS-4a to BI-RADS-3, contingent upon monitoring and safe follow-up, which could serve as an alternative to biopsy. How to ensure more accurate BI-RADS determinations is. Therefore, a key issue affecting the subsequent management of patients (see **Table 2**).

**Table 2:** Interpretation of BI-RADS and data system scores.

BI-RADS	Malignant degree of the lesion	Cancer risk	Patient management
1	Feminine	About 0%	Routine screening
2	Benign	About 0%	Routine screening
3	Possible benign	0% - 2%	6-month short-term interval safety follow-up
4			
4a	Low suspicion of malignancy	2% - 10%	Biopsy
4b	Moderate suspicion of malignancy	10% - 50%	Biopsy
4c	High suspicion of malignancy	50% - 95%	Biopsy
5	Highly suggestive of malignancy	>95%	Biopsy
6	Malignancy	100%	Surgical resection is appropriately based on the patient’s condition

#### 4.1.2 Elastography

Elastography assesses tissue stiffness by applying external forces to deform the tissue, assessing the elastic modulus of the tissue by analyzing the change in ultrasound signal before and after deformation. This provides clearer and more accurate information about the lesions compared to B-US and color Doppler imaging. Elastography has been extensively studied in breast lesion classification. Currently, elastography imaging techniques mainly include strain elastography (SE) and SWE, which may improve the diagnostic specificity of breast ultrasound.

SE reflects tissue stiffness by applying pressure with a probe, observing ultrasound image changes before and after the deformation of the tissue, and calculating the strain ratio or strain rate. Some studies have reported SEN, SPE, and ACC of 0.89, 0.90, and 0.90, respectively, in semiquantitative identifying breast lesions with SE (71). In a meta-analysis of SE and SWE for radiomic-based breast lesion classification, the combined SEN for SE in diagnosing breast lesions was 0.84 (95% CI: 0.82 - 0.87) (72).

SWE mainly uses acoustic radiation force or mechanical vibration to generate shear waves in tissues and detects the propagation velocity of shear waves by ultrasound imaging. The propagation velocity of shear waves is proportional to the elastic modulus of the tissue, so the stiffness of the tissue can be assessed by measuring the shear wave velocity. A meta-analysis on BC diagnosis involving SE and SWE modalities indicated that the combined sensitivity (SEN) for SWE was 0.85 (95% CI: 0.83 - 0.87), underscoring the enhanced diagnostic accuracy when SWE is used in conjunction with other imaging techniques. Although SWE and SE have similar diagnostic efficacy, SWE, with its demonstrated higher reproducibility and objectivity, offers significant practical advantages in medical applications, as evidenced by its successful implementation in assessing liver conditions, diagnosing prostate lesions, and detecting early renal damage in diabetes.

Despite significant advances in elastography for breast lesion classification, several limitations and challenges persist. Elastography results are susceptible to factors such as probe pressure, tissue depth, and respiratory motion. Different brands and models of ultrasound equipment vary in elastography performance, which may lead to inconsistent results. These factors need to be considered in practical applications.

### 4.2 Deep learning application

Traditional ML methods rely on manual feature extraction and visual observation of image morphology by ultrasound workers, which requires domain knowledge and is difficult to capture complex patterns that may have significant inter-observer variations. The application of DL techniques in image classification, object detection, segmentation, and image synthesis provide a new perspective for the diagnosis of breast lesions. CNNs (e.g., ResNet, DenseNet), through the convolution layer CNNs (e.g., ResNet, DenseNet) automatically extract hierarchical image features. Their ability to characterize complex patterns, including subtle findings like architectural distortions and spiculated margins, is significantly superior to traditional methods. However, DL models rely on large-scale labeled data, and the high cost of medical image labeling has led to overfitting in some studies using small samples. The comparison of and advantages and limitations of ML and DL models in breast ultrasound is summarized in **Table 3**. In recent years, with the rapid growth in graphics processor computing power, more and more studies have integrated DL and radiomics (73–75). Approaches that combine DL and radiomics utilize supervised learning techniques. Employing learning methods to preprocess limited image data and derive numerous quantitative features from these images through the machine’s autonomous learning process. The subjectivity and uncertainty of radiologists’ classification choices are reduced to speed up the diagnosis and treatment and liberate the healthcare burden.

**Table 3:** Comparison of traditional machine learning and deep learning in breast ultrasound.

Model	Core features	Feature extraction method	Data requirements	Interpretability	Advantages	Limitations
ML						
SVM	Hyperplane-based classification that relies on manually extracted morphological and textural features	Manual extraction of morphological features (edge regularity, aspect ratio), texture features (gray scale covariance matrix GLCM)	Small samples (<500 cases)	High (based on feature weights)	Fast training for small samples	Reliance on expert characterization makes it difficult to capture complex patterns
RF	Integrated learning approach with voting decisions via multiple decision trees relying on radiomics features	Radiomics characterization (shape, first-order statistics, texture)	Medium sample (500-1000)	Medium (significance of characteristics is open to interpretation)	Robust to high-dimensional data, can be trained in parallel	Characterization works are time-consuming and noise-sensitive
KNN	Sample similarity-based classification, which relies on geometric and echo features, is an inert learning (no explicit training required)	Geometric features (size, circumference), echo characteristics	Small samples	High (based on nearest neighbor samples)	Simple algorithm, no training required	High computational complexity and sensitive to dimensionality
DL						
CNN	Contains a convolutional layer, a pooling layer, and automatically learns hierarchical features from images, and is the underlying architecture for most DL models	Automatically learn multi-level features (from low-level edges to high-level semantics) from raw images	Large samples (>1000 cases)	low	End-to-end learning without manual features	Requires large amounts of labeled data and high training costs
ResNet	The residual connection is introduced on the basis of CNN to solve the problem of deep network training, which belongs to the improved variant of CNN	Introducing residual connectivity to solve the deep network gradient vanishing problem	Medium to large-scale data	low	Trainable Extremely Deep Networks	High model complexity and difficult to interpret
DenseNet	Dense connectivity between layers to enhance feature propagation belongs to the optimized architecture of CNNs and is commonly used for image classification	Dense connectivity improves feature propagation efficiency	Large-scale data	low	High parameter efficiency and feature reusability	Longer training time
3D CNN	Extended to three-dimensional space based on CNN, it is suitable for spatio-temporal feature extraction of 3D ultrasound data	Simultaneous extraction of spatial and temporal dimension features	Multicenter data	low	For 3D ultrasound data, capturing spatial structure	Extremely high data requirements and high consumption of computing resources
RNN	Contains cyclic connections for processing sequence data (e.g., ultrasound video timing information) and belongs to the sequence modeling DL model	Processing sequence data (e.g., dynamic ultrasound video)	Medium-scale data	low	Capturing timing information	Training is unstable, and long sequences are difficult to process
GAN	It consists of a generator and a discriminator for image generation and enhancement, and belongs to the DL model of unsupervised learning	Image enhancement and synthesis (e.g., ultrasound image denoising)	Medium-scale series data	low	Generate high-quality synthetic data	Training instability, risk of mode crash

ML, Machine Learning; DL, Deep Learning; CNN, Convolutional Neural Network; RF, Random Forest; SVM, Support Vector Machine; ResNet, Residual Network; KNN, K-Nearest Neighbors; RNN, Recurrent neural network; GAN, Generative adversarial networks.

DL techniques are employed for fully automated breast density segmentation and classification, with results strongly correlating with radiologists’ manual classification. Zhang et al. aim to investigate whether DL-based radiomics models can improve the diagnostic performance of ultrasound for breast lesion classification. They developed DL radiomics models based on B-US and SWE in terms of breast lesion classification and compared them with quantitative SWE parameters and diagnostic assessments by radiologists. The study found that the area under the ROC curve for both the B-US and SWE-based DL radiomics models in the training cohort was 0.99 (95% CI: 0.99 - 1.00). Both DL radiomics models were more specific than the maximum elasticity parameter in both the training and independent validation cohorts, and the DL radiomics model significantly outperformed the quantitative SWE parameter and the BI-RADS assessment for breast lesion classification. Huang et al. found that the CNN-based tumor identification and grading system had an accuracy BI-RADS-3 of 0.99, BI-RADS-4a of 0.94, BI-RADS-4b of 0.73, BI-RADS-4c of 0.92 (76). Ciritsis et al. similarly classified benign and malignant breast ultrasound image classification by BI-RADS and CNN modeling. CNN modeling accuracy for BI-RADS was slightly higher than that of radiologists (ACC: 0.93 vs 0.92) (77). These studies suggest that the integration of DL-based radiomics methods can mimic the human decision-making process and help improve ultrasound’s ability to classify the benign and malignant nature of breast lesions.

However, these impressive accuracy metrics require cautious interpretation. The aforementioned studies are predominantly retrospective and single-center in design, which may introduce selection bias and limit generalizability. Performance achieved in optimized research environments may degrade significantly when models encounter data from different institutions, ultrasound machines, or patient populations—a key test of real-world utility that many models have not yet passed. Therefore, while DL models show great potential, their clinical maturity and readiness for deployment should be viewed as preliminary, pending validation through robust, prospective, multi-center studies.

While these reported accuracies are promising, they require careful interpretation. The performance is typically achieved in optimized, retrospective research environments using single-center data. A major and frequently under-reported limitation is the potential for significant performance degradation when models encounter data from different institutions, ultrasound machines, or patient populations: a key test of real-world utility that many models have not yet passed.

Consequently, for AI to earn a definitive role in clinical pathways, validation must evolve. Future studies should prioritize prospective, multi-center trials that report not only standard accuracy metrics but also clinically meaningful endpoints. These include the reduction in unnecessary biopsies or short-term follow-ups, changes in radiologists’ diagnostic confidence and inter-reader agreement when using AI assistance, and the system’s impact on overall workflow efficiency.

DL models are also more sensitive for identifying malignancy in non-mass breast lesions that do not strictly meet the BI-RADS definition. Non-mass breast lesions that lack distinctive margins, shape, and typical ultrasound features are more difficult to diagnose. These lesions have higher malignancy rates and poorer prognosis and quality of life for patients. Li et al. demonstrated the MobileNet model for the benign-malignant differentiation of non-mass breast lesions achieved an AUC of 0.84 (95% CI: 0.81 - 0.86) in the test set (78). Another study looked for imaging features associated with non-mass malignant breast lesions in the development dataset by multivariate LR and found that the development dataset in the ultrasound classification system had a higher AUC compared to when it was not applied (0.91 vs 0.95, *P* < 0.05) (79). The development of DL model imaging histology may improve the accuracy of early diagnosis of non-mass breast lesions, ultimately improving patient prognosis.

Furthermore, integrating clinical data and pathological outcomes with pre-treatment ultrasound images can enhance the accuracy of breast lesion classification. This is achieved by leveraging radiomics, which employs deep learning models for classification, in conjunction with pertinent clinical information.

### 4.3 Explainable AI and challenges in clinical decision-making

Currently, AI models based on CNNs such as VGG, ResNet, and DenseNet have been applied to some medical image classification fields, improving the screening accuracy of lung nodules, breast cancer, and other diseases. However, the ethical and visualization problems associated with the “black box” nature of complex models such as deep learning remain to be solved. AI models also face significant generalization challenges in cross-institutional applications. Multicenter studies have shown that device differences, inconsistent annotations, and non-standardized acquisition can all lead to reduced diagnostic accuracy of AI models. Synergistic advancement through domain-adaptive algorithms, federated learning frameworks, and clinically standardized workflows is the key to moving AI from the lab to the clinic, where it is needed. XAI technologies such as Gradient-weighted Class Activation Mapping (Grad-CAM) are beginning to be integrated to enhance the transparency of how models are processed. XAI technologies enable clinicians and researchers to track which features in an ultrasound image affect the model’s predictions and diagnostic decisions, ensuring that decisions are based on relevant image lesion features rather than artifacts in the data. This improves the trust and clinical applicability of AI models. In addition, XAI can identify whether the model is relying on flaws such as incorrect lesion features, helping engineers to improve their algorithms in a timely manner (80).

The XAI technology system is mainly divided into intrinsic and extrinsic interpretability (81). Intrinsic interpretability is based on the structural characteristics of the model, such as the weight coefficients of linear regression, the splitting rule of decision tree, etc., which naturally possesses human-understandable decision logic. Extrinsic interpretability interprets black-box models by means of *post-hoc* analysis, which is mainly run by feature importance analysis, visualization techniques, counterfactual interpretation, etc. SHAP (SHapley Additive exPlanations) is mainly based on the principle of game theory, which quantifies global feature importance by quantifying the contribution of each feature to the model prediction. In interpreting breast cancer AI models, SHAP values can point to a max influence of “number of calcified foci” on malignancy judgments. LIME (Local Interpretable Model-agnostic Explanations) explains local decision logic by generating linear models near the point of prediction. Grad-CAM localizes the region of interest of the CNN model in the image through gradient backpropagation.

While the current use of XAI technology has made model predictions more transparent, helping clinicians validate and trust AI-driven diagnoses. However, the development of medical XAI still faces significant challenges. The difficulty in translating heat map model results to clinical imaging terminology still biases physicians’ trust and use of model results (82).

### 4.4 Integration into clinical workflow and impact on diagnostic pathways

For AI models to realize their potential, diagnostic accuracy is a prerequisite, but their ultimate value will be determined by seamless integration into existing clinical workflows and their tangible impact on patient management decisions. Current evidence supporting such integration and impact remains limited. The implementation of AI in breast ultrasound can be envisioned in several roles: as a concurrent read assistant providing real-time feedback during image acquisition, a second reader for independent verification post-scan, or an automated triage tool flagging high-priority cases. Each mode presents distinct implications for workflow efficiency, radiologist responsibility, and required regulatory approval.

The most significant clinical impact of AI is anticipated in refining the management of diagnostically challenging cases, particularly within the BI-RADS 3 and BI-RADS 4a categories. By supplementing subjective visual assessment with quantitative risk scores, AI has the potential to reduce unnecessary short-term follow-ups for stable, very low-risk BI-RADS 3 lesions, and to decrease benign biopsy rates for BI-RADS 4a lesions by more confidently upgrading a high-risk subset. This optimization of the diagnostic pathway can alleviate patient anxiety, improve healthcare resource allocation, and potentially expedite treatment for aggressive malignancies. A forward-looking step would be the formal exploration of integrating AI-derived quantitative risk scores as a supplementary descriptor within the BI-RADS lexicon, potentially leading to a more nuanced and personalized risk assessment framework.

Successful translation, however, hinges on overcoming significant non-technical barriers that extend far beyond diagnostic accuracy. Clinician trust and acceptance must be cultivated, which depends not only on model interpretability but also on consistent performance within local clinical contexts and clear protocols for reconciling AI suggestions with clinical judgment. Seamless workflow integration requires adapting standardized reporting frameworks to incorporate AI outputs meaningfully, without creating ambiguity or additional burden. Furthermore, navigating the regulatory pathways for AI as a medical device demands a higher level of evidence, typically through prospective trials that demonstrate tangible clinical utility. Perhaps most critically, the widespread adoption of AI necessitates the development of clear medico-legal and ethical frameworks to address accountability, liability, and algorithmic bias, ensuring that these tools are deployed responsibly and equitably. Finally, demonstrating conclusive health economic value through tailored cost-effectiveness analyses is essential for sustainable implementation within diverse healthcare systems. Future development must, therefore, transition from proof-of-concept accuracy studies to pragmatic trials that measure clinically relevant endpoints such as the positive predictive value of AI-guided biopsy recommendations, changes in diagnostic confidence, and time-to-treatment intervals. The in-depth integration of XAI and medical knowledge still has a long way to go, and there is an urgent need to combine text, images, videos, and other forms to improve the intuitiveness of the explanation. Secondly, XAI technology has limitations: SHAP values have global interpretation consistency, but require thousands of model inferences and high computational complexity; LIME provides local interpretability, but may introduce bias due to simplified assumptions; Grad-CAM visualization is intuitive, but can only locate spatial regions and cannot quantify feature contribution. This difference in technical characteristics requires a multi-method joint interpretation strategy for clinical applications.

## 5 Commercial computer-aided detection and diagnostic systems

Radiologists cannot analyze medical image data accurately and completely for long periods due to fatigue, lack of experience, and other factors (83, 84). The application of CAD systems in medical imaging has been vigorously explored and developed, with a history dating back to the 1970s. These systems, leveraging advanced technologies such as computer vision, deep learning, and artificial intelligence, have significantly improved diagnostic accuracy and efficiency. They are now widely used in various medical imaging fields, including CT, MRI, and ultrasound, and are crucial for the early detection and treatment of diseases like cancer. In the context of breast ultrasound, these systems are increasingly positioned not merely as detection tools but as integrated decision-support aids designed to augment the radiologist’s performance, particularly in characterizing indeterminate lesions and reducing perceptual errors. Commercially available CAD systems are now accessible globally, and the subcommittee on CAD in diagnostic imaging aims to integrate the latest technological advancements and developments in CAD into diagnostic practices. Nosis in medical imaging and to develop techniques, practices, and standards to address practical application issues that arise in clinical settings.

CAD can effectively evaluate imaging data and integrate it into statistical algorithm models for analysis, assisting clinical physicians in making accurate diagnoses. CAD systems can provide specific lesion location information and/or diagnostic analysis (85). The computer-aided detection system mainly reminds radiologists to pay attention to these areas by marking image regions that may display abnormal lesions. The computer-aided diagnostic system, combined with other relevant diagnostic data and biomarkers, provides clinical physicians with an assessment of disease type, severity, staging, progression, or resolution.

Commercially available conventional CAD systems consist of localizing the ROI and classifying the ROI for breast lesion classification. Instantaneous results on the nature of the lesions are provided by analyzing the ROI in the form of frozen images on ultrasound devices, either automatically or manually selected. However, due to the complexity of the breast structure and the presence of noise in the ultrasound images, traditional manual characterization methods usually fail to achieve satisfactory results (86). These systems assist radiologists in detecting lesions and differentiating between benign and malignant lesions, serving as a “second opinion” to improve diagnostic accuracy and reduce unnecessary recalls. However, potential radiomic CAD systems warrant further exploration.

### 5.1 S-Detect

S-Detect is regarded as the most crucial image analysis software program for commercial CAD systems for the breast. S-Detect directly pinpoints breast lesions and manually selects ROI to detect lesions on the ultrasound devices even after freezing the image and uploading it to the workstation. The addition of S-Detect clearly distinguishes the features with high malignant representation by outlining the ROI independently and improving the diagnostic efficiency.

S-Detect improves the use of breast ultrasound in clinical practice and assists radiologists in making correct diagnostic decisions. Kiwook K. et al. conducted the first performance study of an AI-driven tool involving 192 breast lesions. They found that S-Detect classified diagnoses with a significantly higher AUC than radiologists (0.73 vs. 0.65, *P* = 0.04). This aligns with broader research indicating AI’s potential to enhance diagnostic accuracy in breast cancer. A study determining the effect of S-Detect on the diagnostic ability of radiologists of different experiences found that the diagnostic accuracy of inexperienced radiologists improved significantly with CAD assistance, as well as the SPE and PPV of experienced radiologists. Diagnostic results between radiologists and S-Detect showed moderate concordance (Kappa = 0.58) (87, 88). Wang et al. demonstrated significant improvement in AUC for radiologists of different experience when using S-Detect for categorical diagnosis (89). S-Detect shows potential to augment diagnostic accuracy, particularly for less-experienced readers. However, its utility is not uniform; performance is highly dependent on the chosen BI-RADS threshold and remains subject to variability from manual ROI selection. Most importantly, its effectiveness and consistency across diverse clinical settings lack robust validation through large-scale, multi-center prospective studies, which is necessary before broad clinical recommendations can be made.

S-Detect also improves inter- and intra-observer concordance (90). The final assessment of inter-observer variability by category for ultrasound was significantly improved (*P* < 0.001) in Park’s study (87).

Different definitions of benign and malignant for BI-RADS may lead to different diagnostic efficacy of S-Detect. The diagnostic efficacy of S-Detect was significantly higher than that of radiologists when the threshold of benignity and malignancy of the lesion was set to the BI-RADS-4a (ACC: 0.71 vs 0.56, *P* < 0.05). However, the diagnostic efficacy of S-Detect was significantly lower than that of radiologists (ACC: 0.71 vs 0.87, *P* < 0.05) when the threshold value was set to BI-RADS-4b (89). At the same time, S-Detect also leads to changes in diagnostic BI-RADS classification (91, 92). However, the diagnostic performance of S-Detect demonstrates significant dependency on BI-RADS threshold definitions. This performance discrepancy underscores potential limitations in risk stratification across BI-RADS categories. Additionally, the requirement for manual ROI selection introduces operator-dependent variability, as inter-operator differences in lesion contouring may influence feature extraction and classification outcomes. Multicenter validation studies are currently lacking to establish the generalizability of diagnostic consistency across diverse clinical settings. The analysis of the benignness or malignancy of BI-RADS lesions by S-Detect remains questionable, and S-Detect does not completely liberate radiologists from ultrasound diagnosis. The stability of the diagnostic efficacy of the S-Detect software for assisted classification of breast lesions under different definitions and its adaptability to the BI-RADS should be continuously explored in clinical practice.

### 5.2 Other systems

In addition to S-Detect software, CAD systems such as MobileNet, KOIOS, and others have shown good value in breast lesion classification.

MobileNet can accurately classify breast lesions, especially BI-RADS-4 breast lesions (93). A screening study involving 479 BI-RADS-4a breast lesions found that the MobileNet model performed best in classification (AUC: 0.90, ACC: 0.91). It was predicted that 14.4% of BI-RADS-4a patients would be upgraded to BI-RADS-4b as a result of MobileNet modeling (94). The good performance of MobileNet for benign and malignant differentiation of non-mass breast lesions has also been described previously (78). Compared with the base MobileNet model, the novel MobileNet-v2 model improved based on super-resolution ultrasound images and demonstrated better diagnostic performance in the breast lesion classification, with its AUC improved by 0.09 and 0.03 in the training and test sets, respectively (95). The MobileNet model can reduce the incidence of malignant tumors in breast lesions diagnosed as BI-RADS-4a on preoperative ultrasound examination, enabling clinicians to strengthen the attention to such patients, make timely and correct management, and avoid delays. Although MobileNet achieves a great AUC for BI-RADS classification, but struggles with non-mass lesions. Transfer learning-based models like MobileNet-v2 require large-scale, standardized datasets to avoid overfitting.

KOIOS DS TM (KOIOS) has demonstrated excellent performance as a new CAD system in providing risk assessment for breast malignancy. KOIOS redefines breast lesion classification based primarily on the BI-RADS (see **Table 4**). KOIOS, like S-Detect, improves the correct assessment of ultrasound breast lesions for most physicians (95). Its decision support for the assessment of breast ultrasound lesions may reduce unnecessary biopsies. KOIOS has a significant impact on the assessment of lesions in the breast and on decisions about whether to biopsy a lesion (96). The SEN of KOIOS for performing breast lesion classification and deciding whether to biopsy a lesion was 0.87 (95% CI: 0.84 - 0.90), whereas the mean SEN for radiologists’ judgments from ultrasound images only was 0.83 (95% CI: 0.78 - 0.89) (97). Patients with KOIOS-recommended biopsies had a higher rate of positivity compared with BI-RADS-recommended biopsies (98). At the same time, KOIOS may also improve inter- and intra-observer agreements. However, it has been criticized for underperforming in patients with dense breast tissue (99). Its reliance on multimodal radiomics may introduce bias when clinical data is incomplete (100).

**Table 4:** KOIOS system rating interpretation.

KOIOS	Cancer risk	BI-RADS
Benign (Kbe)	0% - 0.5%	BI-RADS 1 BI-RADS 2
Possible benign (Kpb)	0.5% - 2%	BI-RADS 3
Suspicious (KSS)	2% - 50%	BI-RADS-4a BI-RADS-4b
Possible malignant (KPM)	50% - 95%	BI-RADS-4c BI-RADS 5

Despite the vigorous development and achievements of S-Detect and KOIOS software, CAD systems for BC screening continue to face numerous challenges, notably the absence of a global public dataset, complexities in binary classification, inadequate image quality, and a heavy reliance on manual ROI annotation. There is still a long way to go in the development of commercial CAD systems.

## 6 Evaluating translational readiness: bridging the gap between research and clinic

The transition of AI from research prototypes to reliable clinical tools necessitates a critical appraisal of their validation rigor and real-world robustness. **Table 5** provides a systematic comparison of key characteristics between typical single-center research prototypes and commercially available multi-center validated systems, highlighting the gaps that must be addressed for widespread adoption.

**Table 5:** Systematic comparison between single-center research prototypes and commercially deployed AI systems in breast ultrasound.

Aspect	Research prototype	Commercial system	Core translational gap
Data & Validation	Single-center, retrospective. Internal validation	Multi-center, prospective. External validation required	Proven on local data vs. validated for broad use
Performance Focus	Aggregate metrics	Clinical endpoint impact	Benchmark excellence vs. clinical utility
Failure & Robustness	Rarely analyzed	Failure mode analysis is mandated	Unknown reliability vs. quantified safety
Development Cycle	Fast, novel algorithms	Slow, regulated updates	Pursuit of novelty vs. requirement for stability
Success Metric	Algorithmic novelty, benchmark rank	Clinical adoption, cost-benefit	Academic achievement vs. healthcare impact

### 6.1 The single-center research paradigm: high performance with limited generalizability

Studies developing novel AI models (e.g., customized CNNs, GANs for enhancement) predominantly follow a single-center, retrospective design. While these works demonstrate impressive accuracy (often AUC >0.90) and drive methodological innovation, their results are intrinsically tied to the local data domain. Performance claims are vulnerable to domain shift and may not hold across different ultrasound machines, patient demographics, or institutional practices. The primary value of this paradigm lies in proof-of-concept and algorithm advancement, not in proving clinical readiness.

### 6.2 The commercial CAD pathway: emphasis on standardization and multi-center assessment

Commercial systems like S-Detect and KOIOS DS represent a later stage in the translational pipeline. Their development increasingly incorporates data from multiple sites, albeit often under controlled conditions. More importantly, their evaluation increasingly includes multi-center validation studies (e.g., references 87, 97). While reported diagnostic metrics may sometimes appear more modest than research prototypes, they often reflect performance across a broader range of clinical scenarios. The focus shifts towards workflow integration, user interface design, regulatory clearance, and demonstrating consistent utility across diverse settings—key factors for clinical adoption that are frequently absent in research papers.

### 6.3 The critical role of external validation and handling heterogeneity

The most significant distinction between a research prototype and a clinically viable tool is the evidence from prospective, externally validated trials. Future research must prioritize this step. Furthermore, explicit strategies to mitigate domain shift are needed. These include: (1) Developing and reporting model performance stratified by ultrasound device vendor or model; (2) Utilizing domain adaptation techniques in model training to improve robustness to acquisition differences; (3) Federated learning frameworks that enable training on distributed data without centralizing sensitive patient information, thereby inherently encompassing multi-center heterogeneity.

## 7 Conclusions and future perspectives

**Figure 3:**
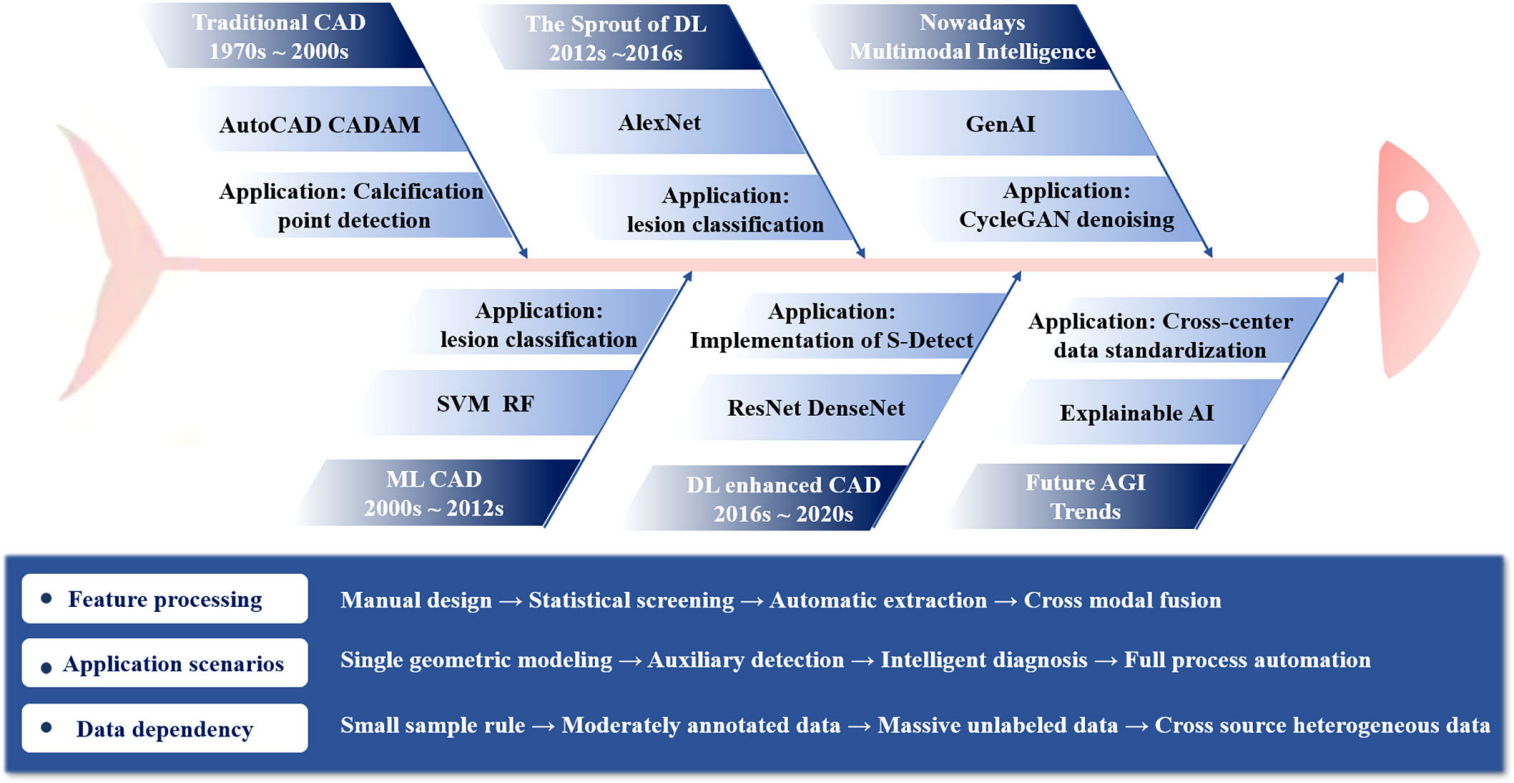
Evolution from traditional CAD to DL-enhanced systems.

This narrative review comprehensively examines recent advancements in AI for breast ultrasound, with a critical focus on its evolving role in lesion classification and the significant translational gaps that must be addressed. Selected studies under controlled, often single-center retrospective conditions have demonstrated that AI models can achieve high classification accuracy for specific tasks. A roadmap diagram showing evolution from traditional CAD to DL enhanced systems has been clearly presented in **Figure 3**. However, it is imperative to contextualize these figures. They predominantly originate from single-center, retrospective evaluations often conducted on curated datasets. The translational leap to reliable, generalizable performance in heterogeneous clinical practice—where image quality, patient demographics, and equipment vary widely—remains largely unproven and constitutes the foremost current challenge. Within optimized research settings, the advancement of AI technology has demonstrated the potential to significantly improve the precision and efficiency of diagnosing breast diseases, as evidenced by studies showing AI’s ability to enhance the accuracy of BC detection and reduce the workload of radiologists (see **Table 6**). However, the reproducibility of these benefits across diverse clinical environments awaits confirmation through more rigorous validation. Furthermore, we underscore the paramount importance of radiomics in breast lesion classification and clinical practice, to provide radiologists and clinicians with theoretical support for diagnosis and treatment. The integration of radiomics into the clinical practice of breast lesion staging is expected to enhance the efficiency of early screening and facilitate timely diagnosis and treatment of breast cancer. The integration of explainable AI and strategies to improve cross-institutional generalization will be pivotal for translating AI from research to clinical practice. However, realizing this potential requires more than technical excellence; it demands concerted efforts in addressing data heterogeneity across institutions, establishing robust regulatory pathways for AI as a medical device, and fostering interdisciplinary collaboration among engineers, clinicians, and healthcare administrators.

**Table 6:** AI Models for breast lesion classification in ultrasound.

Study	Year	AI model	Dataset	Sample size	Objective	Performance metrics
Wan KW et al. (33)	2021	AutoML Vision	Cairo University Breast Ultrasound Images dataset Mendeley Data BUS dataset	895 images	Breast Lesions Classification	The best-performing AI model: Random Forest: ACC: 0.90, SEN: 0.71, SPE: 1.00, F1: 0.83, AUCPR: 0.90 Target model: AutoML Vision: ACC: 0.86, SEN: 0.84, SPE: 0.88, F1: 0.83, AUCPR: 0.95
Becker AS et al. (50)	2018	ResNet-50	Private Hospital Dataset	632 cases	Breast Lesions Classification	AUC: 0.84, SEN:0.84, SPE:0.80
Qian X, et al. (51)	2020	A CNN model combining B-mode and color Doppler	Private Hospital Dataset	106565 images	Breast Lesions Classification	AUC: 0.98
Qian L et al. (54)	2021	Res Net and VGG Net	Private Hospital Dataset	320 images	Predict postoperative upgrading of pure ductal carcinoma *in situ* diagnosed by core needle biopsy before surgery.	AUC: 0.76 ACC: 0.74 SEN: 0.73 SPE: 0.75
Cao Z et al. (59)	2019	AlexNet, ZFNet, VGG, ResNet, GoogLeNet and DenseNet	Private Hospital Dataset	1043 cases	Breast lesion detection and classification	The best-performing AI model: ResNet: ACC: 0.75
Balasubramaniam S et al. (60)	2021	LeNet	Kaggle Dataset	971 images	Breast lesion classification	ACC: 0.90
AlZoubi A et al. (61)	2024	BNet, GNet, SqNet, DsNet, RsNet and IncReNet	Private Hospital Dataset	3034 images	Breast lesion classification	The best-performing AI model: BONet: ACC: 0.83
Zhao G et al. (62)	2023	DL-video and DL-image	Private Hospital Dataset	1000 cases	Breast lesion classification	DL-video: AUC: 0.97 DL-image: AUC: 0.93
Huang Y et al. (76)	2019	ROI-CNN	Private Hospital Dataset	2238 images	Breast lesion detection and classification	ACC: 0.99
Ciritsis A et al. (77)	2019	dCNN	Private Hospital Dataset	582 cases	Breast lesion classification	The classification of BI-RADS 2 versus BI-RADS 3-5, ACC: 0.87 The classification of BI-RADS 2–3 versus BI-RADS 4-5, ACC: 0.93 The classification of BI-RADS 2–4 versus BI-RADS 5, ACC: 0.84
Li G et al. (78)	2023	DenseNet 121448 and MobileNet 448	Private Hospital Dataset	824 cases	Breast lesion detection and classification	The better-performing AI model: MobileNet 448: Detection: AUC: 0.99, ACC: 0.97, SEN: 0.97 Classification: AUC: 0.84, ACC: 0.71, SEN: 0.80
Park KW et al. (79)	2021	A classification system	Private Hospital Dataset	715 cases	Breast lesion classification	AUC: 0.95
Park HJ et al. (87)	2017	S-Detect	Private Hospital Dataset	192 cases	Breast lesion classification	When the cutoff was set at category 4a, S-Detect AUC: 0.73, Radiologist AUC: 0.65
Zhao Z et al. (94)	2022	MobileNet, DenseNet 121, Xception and Inception V3	Private Hospital Dataset	479 cases	Breast lesion classification	The best-performing AI model: Mobile Net: AUC:0.90, ACC: 0.91, SEN: 0.93
Yang L.et al. (95)	2023	ORResNet 101, ORMobileNet v2, SRResNet 101, SRMobileNet v2, ORLR, ORSVM, SRLR, SRSVM\	Private Hospital Dataset	333 cases	Breast lesion classification	The best-performing AI model: SRMobileNet v2: AUC improvements of 0.089 and 0.031 in the training and testing sets
Mango VL et al. (97)	2025	Koios DS	Private Hospital Dataset	222 cases	Breast lesion classification	The SEN of KOIOS in classifying breast masses and deciding whether to perform a biopsy on lesions is 0.87

The development of AI requires continuous optimization of validation strategies and rigorous validation. Establishing the reliability of AI models requires a hierarchy of evidence, with prospective external validation on fully independent, multi-center cohorts being the gold standard for assessing real-world generalizability. This process must explicitly account for and analyze performance across different ultrasound equipment vendors and acquisition protocols to quantify and address domain shift. Furthermore, systematic comparisons between research-grade prototypes and commercially deployed systems reveal that readiness for clinical application is determined less by peak accuracy in constrained settings and more by proven robustness, standardized evaluation, and successful integration into heterogeneous clinical environments.

While **Figure 3** and **Table 6** illustrate the technological evolution and performance landscape, translating this potential into routine clinical practice requires a focused roadmap addressing the following critical pathways:

### 7.1 Development of standardized, multi-vendor datasets and benchmarks

The creation of large-scale, publicly available datasets, acquired from diverse ultrasound systems and adhering to standardized imaging protocols, is a fundamental prerequisite. These should serve not only for training but as independent benchmark platforms to rigorously evaluate model generalizability and resilience to domain shift:a key limitation highlighted in current research.

### 7.2 Conducting regulatory-grade clinical trials

Future validation must transition from retrospective accuracy studies to prospective, multi-center trials designed with regulatory endpoints for Software as a Medical Device. These trials should demonstrate improvement in tangible clinical outcomes, such as increased positive predictive value of biopsies or reduced time to definitive diagnosis, providing the evidence base required for FDA, CE, or NMPA clearance.

### 7.3 Formal integration into clinical reporting and decision pathways

For seamless adoption, AI’s role must be defined within the radiological workflow. Its output requires structured integration into the BI-RADS reporting framework. This could involve providing a quantitative malignancy risk score alongside the BI-RADS category or offering decision-support prompts to aid in the management of equivocal lesions, thereby directly influencing patient management algorithms.

Beyond technical and clinical validation, the responsible integration of AI into healthcare demands proactive engagement with its broader regulatory, ethical, and legal ecosystem. Future development must align with evolving standards for Software as a Medical Device, requiring early dialogue with regulatory bodies and study designs that meet approval requirements. Concurrently, the field must establish ethical guidelines and governance structures to ensure fairness, transparency, and accountability in algorithm development and deployment. Clear medico-legal protocols are also needed to define the standard of care and shared responsibility in AI-assisted diagnosis, addressing critical questions of liability and data privacy. Navigating these complex dimensions is not ancillary but fundamental to building the trust required for AI to become a viable and sustained component of clinical practice.

### 7.4 Implementation science and health economics research

Beyond technical and clinical validation, successful deployment necessitates implementation science studies to identify barriers to adoption and robust health economic analyses to demonstrate cost-effectiveness within specific healthcare systems. This evidence is crucial for stakeholder buy-in and sustainable integration.

In conclusion, the journey from promising research to routine practice hinges on concerted efforts across multiple interconnected domains: the creation of standardized, multi-vendor benchmarks; the generation of prospective, regulatory-grade clinical evidence; the development of frameworks for structured clinical integration; the proactive navigation of regulatory, ethical, and legal landscapes; and the demonstration of practical implementation value through health economics research. By prioritizing these comprehensive translational imperatives, the field can ensure that AI evolves into a reliable, trusted, and equitably deployed standard of care in breast ultrasound.

## References

[1] Irmici G Cè M Pepa GD D’Ascoli E De Berardinis C Giambersio E . Exploring the potential of artificial intelligence in breast ultrasound. Crit Rev Oncog. (2024) 29:15–28. doi: 10.1615/critrevoncog.2023048873, PMID: 38505878

[2] Bray F Laversanne M Sung H Ferlay J Siegel RL Soerjomataram I . Global cancer statistics 2022: GLOBOCAN estimates of incidence and mortality worldwide for 36 cancers in 185 countries. CA: Cancer J Clin. (2024) 74:229–63. doi: 10.3322/caac.21834, PMID: 38572751

[3] Burstein HJ Curigliano G Thürlimann B Weber WP Poortmans P Regan MM . Customizing local and systemic therapies for women with early breast cancer: the St. Gallen International Consensus Guidelines for treatment of early breast cancer 2021. Ann Oncol Off J Eur Soc Med Oncol. (2021) 32:1216–35. doi: 10.1016/j.annonc.2021.06.023, PMID: 34242744 PMC9906308

[4] Barzaman K Karami J Zarei Z Hosseinzadeh A Kazemi MH Moradi-Kalbolandi S . Breast cancer: Biology, biomarkers, and treatments. Int Immunopharmacol. (2020) 84:106535. doi: 10.1016/j.intimp.2020.106535, PMID: 32361569

[5] Peart O . Metastatic breast cancer. Radiol Technol. (2017) 88:519m–39m. doi: 10.1016/j.intimp.2020.106535, PMID: 28500107

[6] Xu Y Gong M Wang Y Yang Y Liu S Zeng Q . Global trends and forecasts of breast cancer incidence and deaths. Sci Data. (2023) 10:334. doi: 10.1038/s41597-023-02253-5, PMID: 37244901 PMC10224917

[7] Thigpen D Kappler A Brem R . The role of ultrasound in screening dense breasts-A review of the literature and practical solutions for implementation. Diagnost (Basel Switzerland). (2018) 8. doi: 10.3390/diagnostics8010020, PMID: 29547532 PMC5872003

[8] Yang L Wang S Zhang L Sheng C Song F Wang P . Performance of ultrasonography screening for breast cancer: a systematic review and meta-analysis. BMC Cancer. (2020) 20:499. doi: 10.1186/s12885-020-06992-1, PMID: 32487106 PMC7268243

[9] Giambersio E Depretto C Trimboli RM Di Leo G D’Ascoli E Della Pepa G . Utility of detection of breast calcifications with integrated real-time radiography system (IRRS) during digital breast tomosynthesis (DBT)-guided vacuum assisted biopsy (VAB): initial single-center experience. La Radiol Medica. (2023) 128:699–703. doi: 10.1007/s11547-023-01636-3, PMID: 37115391

[10] Pishdad P Moosavi A Jalli R Zarei F Saeedi-Moghadam M Zeinali-Rafsanjani B . How can additional ultrasonography screening improve the detection of occult breast cancer in women with dense breasts? Polish J Radiol. (2020) 85:e353–e60. doi: 10.5114/pjr.2020.97944, PMID: 32817768 PMC7425225

[11] Zanotel M Bednarova I Londero V Linda A Lorenzon M Girometti R . Automated breast ultrasound: basic principles and emerging clinical applications. La Radiol Medica. (2018) 123:1–12. doi: 10.1007/s11547-017-0805-z, PMID: 28849324

[12] Gu J Jiang T . Ultrasound radiomics in personalized breast management: Current status and future prospects. Front Oncol. (2022) 12:963612. doi: 10.3389/fonc.2022.963612, PMID: 36059645 PMC9428828

[13] Romeo V Cuocolo R Apolito R Stanzione A Ventimiglia A Vitale A . Clinical value of radiomics and machine learning in breast ultrasound: a multicenter study for differential diagnosis of benign and Malignant lesions. Eur Radiol. (2021) 31:9511–9. doi: 10.1007/s00330-021-08009-2, PMID: 34018057 PMC8589755

[14] Mahant SS Varma AR . Artificial intelligence in breast ultrasound: the emerging future of modern medicine. Cureus. (2022) 14:e28945. doi: 10.7759/cureus.28945, PMID: 36237807 PMC9547651

[15] Afrin H Larson NB Fatemi M Alizad A . Deep learning in different ultrasound methods for breast cancer, from diagnosis to prognosis: current trends, challenges, and an analysis. Cancers. (2023) 15. doi: 10.3390/cancers15123139, PMID: 37370748 PMC10296633

[16] Brunetti N Calabrese M Martinoli C Tagliafico AS . Artificial intelligence in breast ultrasound: from diagnosis to prognosis-A rapid review. Diagnost (Basel Switzerland). (2022) 13. doi: 10.3390/diagnostics13010058, PMID: 36611350 PMC9818181

[17] Leung JH Karmakar R Mukundan A Thongsit P Chen MM Chang WY . Systematic meta-analysis of computer-aided detection of breast cancer using hyperspectral imaging. Bioeng (Basel). (2024) 11:1060. doi: 10.3390/bioengineering11111060, PMID: 39593720 PMC11591395

[18] Hsu H Lee KH Karmakar R Mukundan A Attar RS Liu PH . From innovation to application: can emerging imaging techniques transform breast cancer diagnosis? Diagnost (Basel). (2025) 15:2718. doi: 10.3390/diagnostics15212718, PMID: 41226009 PMC12610140

[19] Karmakar R Nagisetti Y Mukundan A Wang HC . Impact of the family and socioeconomic factors as a tool of prevention of breast cancer. World J Clin Oncol. (2025) 16:106569. doi: 10.5306/wjco.v16.i5.106569, PMID: 40503398 PMC12149816

[20] Mintz Y Brodie R . Introduction to artificial intelligence in medicine. Minimally Invasive Ther Allied Technol: MITAT: Off J Soc Minimally Invasive Ther. (2019) 28:73–81. doi: 10.1080/13645706.2019.1575882, PMID: 30810430

[21] Gupta R Srivastava D Sahu M Tiwari S Ambasta RK Kumar P . Artificial intelligence to deep learning: machine intelligence approach for drug discovery. Mol Divers. (2021) 25:1315–60. doi: 10.1007/s11030-021-10217-3, PMID: 33844136 PMC8040371

[22] Yu KH Healey E Leong TY Kohane IS Manrai AK . Medical artificial intelligence and human values. New Engl J Med. (2024) 390:1895–904. doi: 10.1056/nejmra2214183, PMID: 38810186 PMC12425466

[23] Lai YC Chen HH Hsu JF Hong YJ Chiu TT Chiou HJ . Evaluation of physician performance using a concurrent-read artificial intelligence system to support breast ultrasound interpretation. Breast (Edinburgh Scotland). (2022) 65:124–35. doi: 10.1016/j.breast.2022.07.009, PMID: 35944352 PMC9379669

[24] Masters K Herrmann-Werner A Festl-Wietek T Taylor D . Preparing for artificial general intelligence (AGI) in health professions education: AMEE guide no. 172. Med Teacher. (2024) 46:1258–71. doi: 10.1080/0142159x.2024.2387802, PMID: 39115700

[25] Teo ZL Quek CWN Wong JLY Ting DSW . Cybersecurity in the generative artificial intelligence era. Asia Pacif J Ophthalmol (Philadelphia Pa). (2024) 13:100091. doi: 10.1016/j.apjo.2024.100091, PMID: 39209217

[26] Waqas A Bui MM Glassy EF El Naqa I Borkowski P Borkowski AA . Revolutionizing digital pathology with the power of generative artificial intelligence and foundation models. Lab Investigat J Tech Methods Pathol. (2023) 103:100255. doi: 10.1016/j.labinv.2023.100255, PMID: 37757969

[27] Handelman GS Kok HK Chandra RV Razavi AH Lee MJ Asadi H . eDoctor: machine learning and the future of medicine. J Internal Med. (2018) 284:603–19. doi: 10.1111/joim.12822, PMID: 30102808

[28] Do S Song KD Chung JW . Basics of deep learning: A radiologist’s guide to understanding published radiology articles on deep learning. Korean J Radiol. (2020) 21:33–41. doi: 10.3348/kjr.2019.0312, PMID: 31920027 PMC6960318

[29] Litjens G Kooi T Bejnordi BE Setio AAA Ciompi F Ghafoorian M . A survey on deep learning in medical image analysis. Med Image Anal. (2017) 42:60–88. doi: 10.1016/j.media.2017.07.005, PMID: 28778026

[30] Brattain LJ Telfer BA Dhyani M Grajo JR Samir AE . Machine learning for medical ultrasound: status, methods, and future opportunities. Abdominal Radiol (New York). (2018) 43:786–99. doi: 10.1007/s00261-018-1517-0, PMID: 29492605 PMC5886811

[31] Hubbard AE Kherad-Pajouh S van der Laan MJ . Statistical inference for data adaptive target parameters. Int J Biostat. (2016) 12:3–19. doi: 10.1515/ijb-2015-0013, PMID: 27227715

[32] Choi RY Coyner AS Kalpathy-Cramer J Chiang MF Campbell JP . Introduction to machine learning, neural networks, and deep learning. Trans Vision Sci Technol. (2020) 9:14. doi: 10.1167/tvst.9.2.14, PMID: 32704420 PMC7347027

[33] Wan KW Wong CH Ip HF Fan D Yuen PL Fong HY . Evaluation of the performance of traditional machine learning algorithms, convolutional neural network and AutoML Vision in ultrasound breast lesions classification: a comparative study. Quant Imaging Med Surg. (2021) 11:1381–93. doi: 10.21037/qims-20-922, PMID: 33816176 PMC7930687

[34] Pehrson LM Lauridsen C Nielsen MB . Machine learning and deep learning applied in ultrasound. Ultraschall der Med (Stuttgart Germany: 1980). (2018) 39:379–81. doi: 10.1055/a-0642-9545, PMID: 30071556

[35] Mousavi SM Beroza GC . Deep-learning seismology. Sci (New York NY). (2022) 377:eabm4470. doi: 10.1126/science.abm4470, PMID: 35951699

[36] Kriegeskorte N Golan T . Neural network models and deep learning. Curr Biol: CB. (2019) 29:R231–r6. doi: 10.1016/j.cub.2019.02.034, PMID: 30939301

[37] LeCun Y Bengio Y Hinton G . Deep learning. Nature. (2015) 521:436–44. doi: 10.1038/nature14539, PMID: 26017442

[38] Miotto R Wang F Wang S Jiang X Dudley JT . Deep learning for healthcare: review, opportunities and challenges. Briefings Bioinf. (2018) 19:1236–46. doi: 10.1093/bib/bbx044, PMID: 28481991 PMC6455466

[39] Georgevici AI Terblanche M . Neural networks and deep learning: a brief introduction. Intensive Care Med. (2019) 45:712–4. doi: 10.1007/s00134-019-05537-w, PMID: 30725133

[40] Lee C Kim Y Kim YS Jang J . Automatic disease annotation from radiology reports using artificial intelligence implemented by a recurrent neural network. AJR Am J Roentgenol. (2019) 212:734–40. doi: 10.2214/ajr.18.19869, PMID: 30699011

[41] Alam MS Wang D Sowmya A . AMFP-net: Adaptive multi-scale feature pyramid network for diagnosis of pneumoconiosis from chest X-ray images. Artif Intell Med. (2024) 154:102917. doi: 10.1016/j.artmed.2024.102917, PMID: 38917599

[42] Parsa M Rad HY Vaezi H Hossein-Zadeh GA Setarehdan SK Rostami R . EEG-based classification of individuals with neuropsychiatric disorders using deep neural networks: A systematic review of current status and future directions. Comput Methods Programs Biomed. (2023) 240:107683. doi: 10.1016/j.cmpb.2023.107683, PMID: 37406421

[43] Wenzel M . Generative adversarial networks and other generative models. In: Colliot O , editor. Machine Learning for Brain Disorders. Humana, New York, NY (2023). p. 139–92. doi: 10.1007/978-1-0716-3195-9_5, PMID: 37988513

[44] Dreyer KJ Geis JR . When machines think: radiology’s next frontier. Radiology. (2017) 285:713–8. doi: 10.1148/radiol.2017171183, PMID: 29155639

[45] Avanzo M Wei L Stancanello J Vallières M Rao A Morin O . Machine and deep learning methods for radiomics. Med Phys. (2020) 47:e185–202. doi: 10.1002/mp.13678, PMID: 32418336 PMC8965689

[46] Luijten B Cohen R de Bruijn FJ Schmeitz HAW Mischi M Eldar YC . Adaptive ultrasound beamforming using deep learning. IEEE Trans Med Imaging. (2020) 39:3967–78. doi: 10.1109/tmi.2020.3008537, PMID: 32746139

[47] Liu J Li C Liu L Chen H Han H Zhang B . Speckle noise reduction for medical ultrasound images based on cycle-consistent generative adversarial network. Biomed Signal Process Control. (2023) 86. doi: 10.1109/tbme.2008.923140, PMID: 18713684

[48] Wang Y Ge X Ma H Qi S Zhang G Yao Y . Deep learning in medical ultrasound image analysis: A review. IEEE Access. (2021) 9:54310–24. doi: 10.1109/ACCESS.2021.3071301, PMID: 41116384

[49] Sudharson S Kokil P . Computer-aided diagnosis system for the classification of multi-class kidney abnormalities in the noisy ultrasound images. Comput Methods Programs Biomed. (2021) 205:106071. doi: 10.1016/j.cmpb.2021.106071, PMID: 33887632

[50] Becker AS Mueller M Stoffel E Marcon M Ghafoor S Boss A . Classification of breast cancer in ultrasound imaging using a generic deep learning analysis software: a pilot study. Br J Radiol. (2018) 91:20170576. doi: 10.1259/bjr.20170576, PMID: 29215311 PMC5965470

[51] Qian X Zhang B Liu S Wang Y Chen X Liu J . A combined ultrasonic B-mode and color Doppler system for the classification of breast masses using neural network. Eur Radiol. (2020) 30:3023–33. doi: 10.1007/s00330-019-06610-0, PMID: 32006174

[52] Shin Y Lowerison MR Wang Y Chen X You Q Dong Z . Context-aware deep learning enables high-efficacy localization of high concentration microbubbles for super-resolution ultrasound localization microscopy. Nat Commun. (2024) 15:2932. doi: 10.1038/s41467-024-47154-2, PMID: 38575577 PMC10995206

[53] Dai F Li Y Zhu Y Li B Shi Q Chen Y . B-mode ultrasound to elastography synthesis using multiscale learning. Ultrasonics. (2024) 138:107268. doi: 10.1016/j.ultras.2024.107268, PMID: 38402836

[54] Qian L Lv Z Zhang K Wang K Zhu Q Zhou S . Application of deep learning to predict underestimation in ductal carcinoma in *situ* of the breast with ultrasound. Ann Trans Med. (2021) 9:295. doi: 10.21037/atm-20-3981, PMID: 33708922 PMC7944276

[55] Hahnloser RH Sarpeshkar R Mahowald MA Douglas RJ Seung HS . Digital selection and analogue amplification coexist in a cortex-inspired silicon circuit. Nature. (2000) 405:947–51. doi: 10.1038/35016072, PMID: 10879535

[56] Kaur G Sinha R Tiwari PK Yadav SK Pandey P Raj R . Face mask recognition system using CNN model. Neurosci Inform. (2022) 2:100035. doi: 10.1016/j.neuri.2021.100035, PMID: 36819833 PMC8656214

[57] Chartrand G Cheng PM Vorontsov E Drozdzal M Turcotte S Pal CJ . Deep learning: A primer for radiologists. Radiogr: Rev Publ Radiol Soc North America Inc. (2017) 37:2113–31. doi: 10.1148/rg.2017170077, PMID: 29131760

[58] Singh D Kumar V Kaur M . Densely connected convolutional networks-based COVID-19 screening model. Appl Intell (Dordrecht Netherlands). (2021) 51:3044–51. doi: 10.1007/s10489-020-02149-6, PMID: 34764584 PMC7867501

[59] Cao Z Duan L Yang G Yue T Chen Q . An experimental study on breast lesion detection and classification from ultrasound images using deep learning architectures. BMC Med Imaging. (2019) 19:51. doi: 10.1186/s12880-019-0349-x, PMID: 31262255 PMC6604293

[60] Balasubramaniam S Velmurugan Y Jaganathan D Dhanasekaran S . A modified LeNet CNN for breast cancer diagnosis in ultrasound images. Diagnostics. (2023) 13. doi: 10.3390/diagnostics13172746, PMID: 37685284 PMC10486538

[61] AlZoubi A Lu F Zhu Y Ying T Ahmed M Du H . Classification of breast lesions in ultrasound images using deep convolutional neural networks: transfer learning versus automatic architecture design. Med Biol Eng Comput. (2024) 62:135–49. doi: 10.1007/s11517-023-02922-y, PMID: 37735296 PMC10758370

[62] Zhao G Kong D Xu X Hu S Li Z Tian J . Deep learning-based classification of breast lesions using dynamic ultrasound video. Eur J Radiol. (2023) 165:110885. doi: 10.1016/j.ejrad.2023.110885, PMID: 37290361

[63] Guo DH Lu CY Chen DL Yuan JZ Duan QM Xue Z . A multimodal breast cancer diagnosis method based on Knowledge-Augmented Deep Learning. Biomed Signal Process Control. (2024) 90. doi: 10.1016/j.bspc.2023.105843, PMID: 41727822

[64] Nardone V Reginelli A Grassi R Boldrini L Vacca G D’Ippolito E . Delta radiomics: a systematic review. La Radiol Medica. (2021) 126:1571–83. doi: 10.1007/s11547-021-01436-7, PMID: 34865190

[65] Gillies RJ Kinahan PE Hricak H . Radiomics: images are more than pictures, they are data. Radiology. (2016) 278:563–77. doi: 10.1148/radiol.2015151169, PMID: 26579733 PMC4734157

[66] Lambin P Leijenaar RTH Deist TM Peerlings J de Jong EEC van Timmeren J . Radiomics: the bridge between medical imaging and personalized medicine. Nat Rev Clin Oncol. (2017) 14:749–62. doi: 10.1038/nrclinonc.2017.141, PMID: 28975929

[67] Wang C Zhu X Hong JC Zheng D . Artificial intelligence in radiotherapy treatment planning: present and future. Technol Cancer Res Treat. (2019) 18:1533033819873922. doi: 10.1177/1533033819873922, PMID: 31495281 PMC6732844

[68] Jiang M Li CL Luo XM Chuan ZR Lv WZ Li X . Ultrasound-based deep learning radiomics in the assessment of pathological complete response to neoadjuvant chemotherapy in locally advanced breast cancer. Eur J Cancer (Oxford England: 1990). (2021) 147:95–105. doi: 10.1016/j.ejca.2021.01.028, PMID: 33639324

[69] Qi YJ Su GH You C Zhang X Xiao Y Jiang YZ . Radiomics in breast cancer: Current advances and future directions. Cell Rep Med. (2024) 5:101719. doi: 10.1016/j.xcrm.2024.101719, PMID: 39293402 PMC11528234

[70] Magny SJ Shikhman R Keppke AL . Breast imaging reporting and data system. In: StatPearls. StatPearls Publishing LLC (2024). Available online at: https://www.ncbi.nlm.nih.gov/books/NBK459169/. 29083600

[71] Bi WL Hosny A Schabath MB Giger ML Birkbak NJ Mehrtash A . Artificial intelligence in cancer imaging: Clinical challenges and applications. CA: Cancer J Clin. (2019) 69:127–57. doi: 10.3322/caac.21552, PMID: 30720861 PMC6403009

[72] Wu H Wang C An Q Qu X Wu X Yan Y . Comparing the accuracy of shear wave elastography and strain elastography in the diagnosis of breast tumors: A systematic review and meta-analysis. Medicine. (2022) 101:e31526. 36343055 10.1097/MD.0000000000031526 PMC9646582

[73] Zheng X Yao Z Huang Y Yu Y Wang Y Liu Y . Deep learning radiomics can predict axillary lymph node status in early-stage breast cancer. Nat Commun. (2020) 11:1236. doi: 10.1038/s41467-020-15027-z, PMID: 32144248 PMC7060275

[74] Huang Y Yao Z Li L Mao R Huang W Hu Z . Deep learning radiopathomics based on preoperative US images and biopsy whole slide images can distinguish between luminal and non-luminal tumors in early-stage breast cancers. EBioMedicine. (2023) 94:104706. doi: 10.1016/j.ebiom.2023.104706, PMID: 37478528 PMC10393555

[75] Liu H Zou L Xu N Shen H Zhang Y Wan P . Deep learning radiomics based prediction of axillary lymph node metastasis in breast cancer. NPJ Breast Cancer. (2024) 10:22. doi: 10.1038/s41523-024-00628-4, PMID: 38472210 PMC10933422

[76] Huang Y Han L Dou H Luo H Yuan Z Liu Q . Two-stage CNNs for computerized BI-RADS categorization in breast ultrasound images. Biomed Eng Online. (2019) 18:8. doi: 10.1186/s12938-019-0626-5, PMID: 30678680 PMC6346503

[77] Ciritsis A Rossi C Eberhard M Marcon M Becker AS Boss A . Automatic classification of ultrasound breast lesions using a deep convolutional neural network mimicking human decision-making. Eur Radiol. (2019) 29:5458–68. doi: 10.1007/s00330-019-06118-7, PMID: 30927100

[78] Li G Tian H Wu H Huang Z Yang K Li J . Artificial intelligence for non-mass breast lesions detection and classification on ultrasound images: a comparative study. BMC Med Inf Decision Making. (2023) 23:174. doi: 10.1186/s12911-023-02277-2, PMID: 37667320 PMC10476370

[79] Park KW Park S Shon I Kim MJ Han BK Ko EY . Non-mass lesions detected by breast US: stratification of cancer risk for clinical management. Eur Radiol. (2021) 31:1693–706. doi: 10.1007/s00330-020-07168-y, PMID: 32888070

[80] Neri E Aghakhanyan G Zerunian M Gandolfo N Grassi R Miele V . Explainable AI in radiology: a white paper of the Italian Society of Medical and Interventional Radiology. La Radiol Medica. (2023) 128:755–64. doi: 10.1007/s11547-023-01634-5, PMID: 37155000 PMC10264482

[81] Sheu RK Pardeshi MS . A survey on medical explainable AI (XAI): recent progress, explainability approach, human interaction and scoring system. Sens (Basel). (2022) 22. doi: 10.3390/s22208068, PMID: 36298417 PMC9609212

[82] Yan M He D Sun Y Huang L Cai L Wang C . Comparative analysis of nomogram and machine learning models for predicting axillary lymph node metastasis in early-stage breast cancer: A study on clinically and ultrasound-negative axillary cases across two centers. Ultrasound Med Biol. (2025) 51:463–74. doi: 10.1016/j.ultrasmedbio.2024.11.003, PMID: 39627056

[83] Renfrew DL Franken EA Jr. Berbaum KS Weigelt FH Abu-Yousef MM . Error in radiology: classification and lessons in 182 cases presented at a problem case conference. Radiology. (1992) 183:145–50. doi: 10.1148/radiology.183.1.1549661, PMID: 1549661

[84] Berbaum KS Franken EA Jr. Dorfman DD Rooholamini SA Kathol MH Barloon TJ . Satisfaction of search in diagnostic radiology. Invest Radiol. (1990) 25:133–40. doi: 10.1097/00004424-199002000-00006, PMID: 2312249

[85] Petrick N Sahiner B Armato SG 3rd Bert A Correale L Delsanto S . Evaluation of computer-aided detection and diagnosis systems. Med Phys. (2013) 40:087001. doi: 10.1118/1.4816310, PMID: 23927365 PMC4108682

[86] Jiang J Jiang X Xu L Zhang Y Zheng Y Kong D . Noise-robustness test for ultrasound breast nodule neural network models as medical devices. Front Oncol. (2023) 13:1177225. doi: 10.3389/fonc.2023.1177225, PMID: 37427110 PMC10325648

[87] Park HJ Kim SM La Yun B Jang M Kim B Jang JY . A computer-aided diagnosis system using artificial intelligence for the diagnosis and characterization of breast masses on ultrasound: Added value for the inexperienced breast radiologist. Medicine. (2019) 98:e14146. doi: 10.1097/md.0000000000014146, PMID: 30653149 PMC6370030

[88] Kim K Song MK Kim EK Yoon JH . Clinical application of S-Detect to breast masses on ultrasonography: a study evaluating the diagnostic performance and agreement with a dedicated breast radiologist. Ultrasonography (Seoul Korea). (2017) 36:3–9. doi: 10.14366/usg.16012, PMID: 27184656 PMC5207353

[89] Wang Y Jiang S Wang H Guo YH Liu B Hou Y . CAD algorithms for solid breast masses discrimination: evaluation of the accuracy and interobserver variability. Ultrasound Med Biol. (2010) 36:1273–81. doi: 10.1016/j.ultrasmedbio.2010.05.010, PMID: 20691917

[90] Lee YJ Choi SY Kim KS Yang PS . Variability in observer performance between faculty members and residents using breast imaging reporting and data system (BI-RADS)-ultrasound, fifth edition (2013). Iranian J Radiol: Q J Published by Iranian Radiol Society. (2016) 13:e28281. doi: 10.5812/iranjradiol.28281, PMID: 27853492 PMC5106650

[91] Bartolotta TV Orlando A Cantisani V Matranga D Ienzi R Cirino A . Focal breast lesion characterization according to the BI-RADS US lexicon: role of a computer-aided decision-making support. La Radiol Medica. (2018) 123:498–506. doi: 10.1007/s11547-018-0874-7, PMID: 29569216

[92] Buchbinder SS Leichter IS Lederman RB Novak B Bamberger PN Sklair-Levy M . Computer-aided classification of BI-RADS category 3 breast lesions. Radiology. (2004) 230:820–3. doi: 10.1148/radiol.2303030089, PMID: 14739315

[93] Gómez-Flores W Coelho de Albuquerque Pereira W . A comparative study of pre-trained convolutional neural networks for semantic segmentation of breast tumors in ultrasound. Comput Biol Med. (2020) 126:104036. doi: 10.1016/j.compbiomed.2020.104036, PMID: 33059238

[94] Zhao Z Hou S Li S Sheng D Liu Q Chang C . Application of deep learning to reduce the rate of Malignancy among BI-RADS 4A breast lesions based on ultrasonography. Ultrasound Med Biol. (2022) 48:2267–75. doi: 10.1016/j.ultrasmedbio.2022.06.019, PMID: 36055860

[95] Yang L Ma Z . Nomogram based on super-resolution ultrasound images outperforms in predicting benign and Malignant breast lesions. Breast Cancer (Dove Med Press). (2023) 15:867–78. doi: 10.2147/bctt.s435510, PMID: 38074418 PMC10700043

[96] Pesapane F Codari M Sardanelli F . Artificial intelligence in medical imaging: threat or opportunity? Radiologists again at the forefront of innovation in medicine. Eur Radiol Experiment. (2018) 2:35. doi: 10.1186/s41747-018-0061-6, PMID: 30353365 PMC6199205

[97] Mango VL Sun M Wynn RT Ha R . Should we ignore, follow, or biopsy? Impact of artificial intelligence decision support on breast ultrasound lesion assessment. AJR Am J Roentgenol. (2020) 214:1445–52. doi: 10.2214/ajr.19.21872, PMID: 32319794 PMC8162774

[98] Browne JL Pascual M Perez J Salazar S Valero B Rodriguez I . AI: can it make a difference to the predictive value of ultrasound breast biopsy? Diagnost (Basel Switzerland). (2023) 13. doi: 10.3390/diagnostics13040811, PMID: 36832299 PMC9955683

[99] Cerdas MG Farhat J Elshafie SI Mariyam F James L Qureshi AK . Exploring the evolution of breast cancer imaging: A review of conventional and emerging modalities. Cureus. (2025) 17:e82762. doi: 10.7759/cureus.82762, PMID: 40416096 PMC12098770

[100] Liu Y Wang Y Hu X Wang X Xue L Pang Q . Multimodality deep learning radiomics predicts pathological response after neoadjuvant chemoradiotherapy for esophageal squamous cell carcinoma. Insights Imaging. (2024) 15:277. doi: 10.1186/s13244-024-01851-0, PMID: 39546168 PMC11568088

